# Apolipoprotein E Genotype Moderation of the Association Between Physical Activity and Brain Health. A Systematic Review and Meta-Analysis

**DOI:** 10.3389/fnagi.2021.815439

**Published:** 2022-01-28

**Authors:** Andrew M. Pearce, Calum Marr, Michaela Dewar, Alan J. Gow

**Affiliations:** Department of Psychology, Centre for Applied Behavioural Sciences, Heriot-Watt University, Edinburgh, United Kingdom

**Keywords:** Alzheimer's disease, apolipoprotein E, brain function, brain structure, lipid profile, meta-analysis, physical activity

## Abstract

**Introduction:**

Possession of one or two e4 alleles of the apolipoprotein E (*APOE*) gene is associated with cognitive decline and dementia risk. Some evidence suggests that physical activity may benefit carriers of the e4 allele differently.

**Method:**

We conducted a systematic review and meta-analysis of studies which assessed *APOE* differences in the association between physical activity and: lipid profile, Alzheimer's disease pathology, brain structure and brain function in healthy adults. Searches were carried out in PubMed, SCOPUS, Web of Science and PsycInfo.

**Results:**

Thirty studies were included from 4,896 papers screened. Carriers of the e4 allele gained the same benefit from physical activity as non-carriers on most outcomes. For brain activation, e4 carriers appeared to gain a greater benefit from physical activity on task-related and resting-state activation and resting-state functional connectivity compared to non-carriers. *Post-hoc* analysis identified possible compensatory mechanisms allowing e4 carriers to maintain cognitive function.

**Discussion:**

Though there is evidence suggesting physical activity may benefit e4 carriers differently compared to non-carriers, this may vary by the specific brain health outcome, perhaps limited to brain activation. Further research is required to confirm these findings and elucidate the mechanisms.

## Introduction

While research has supported the potential benefit of physical activity across a range of cognitive and brain health outcomes, there are indications that not all individuals experience this to the same extent. Possession of the e4 allele of the apolipoprotein E (*APOE*) gene, a risk factor for cognitive decline and dementia, may moderate the association between physical activity and brain health. Research has suggested that individuals possessing the e4 allele may actually benefit more from physical activity, compared to non-carriers. However, findings are variable, both across individual studies and the brain health outcomes considered. The current systematic review explored whether *APOE* moderated the association between physical activity and brain health, including specific cardiovascular or cerebrovascular markers implicated in the mechanisms.

### Physical Activity and Brain Health

Understanding how lifestyle affects the brain is crucial for maintaining our cognitive abilities as we get older. Even in the absence of any diagnosed cognitive impairment, cognitive abilities follow different trajectories through the lifespan. The typical progression involves relative stability or slight increases from our mid-twenties through to our fifties, followed by a gradual decline from our sixties (Schaie et al., 2004). A similar pattern can be seen for brain structure and health (Vinke et al., 2018).

Physical activity is a modifiable lifestyle factor associated with preserved cognitive ability (Erickson et al., 2019). Encouragingly, randomised controlled trials suggest a causative role, with physical activity interventions resulting in improved cognitive performance. For example, executive function (Stern et al., 2019) and spatial memory (Erickson et al., 2011) improved in those undertaking an aerobic exercise intervention compared to a control group engaging only in stretching exercises. Physical activity may also predict future cognitive change. In a longitudinal study which assessed cognitive ability four times between the ages of 79 and 90, greater physical activity undertaken between the ages of 60 and 75 was associated with less cognitive decline over the 11-year period (Gow et al., 2017).

The mechanisms through which physical activity benefits cognition may involve a range of physiological and brain health outcomes. One part of this mechanism is cholesterol, which is transported in the blood by lipoproteins. Higher low density lipoprotein cholesterol (LDL), often referred to as “bad cholesterol,” indicates surplus cholesterol in the blood. In contrast, “good” high density lipoprotein cholesterol (HDL) transports cholesterol back to the liver for disposal. Due to the different effects of LDL and HDL, combined measurements of total cholesterol (TC) can be misleading (Mann et al., 2014). However, assessments of LDL and HDL separately demonstrate a clear association between physical activity and lipid profile, with physically active individuals having reduced LDL (Sarzynski et al., 2015) and increased HDL (Thompson et al., 1997; Kodama et al., 2007).

Physical activity may also be associated with Alzheimer's disease (AD) pathology. The neuropathological hallmarks of AD are senile plaques that contain amyloid beta (Aβ) and intracellular neurofibrillary tangles which consist of tau proteins. Higher levels of brain Aβ are associated with poorer cognitive ability and increased risk of dementia. The most reliable measurement of Aβ is with a tracer such as Pittsburgh compound B (PiB) during positron emission tomography (PET). In a cross-sectional study, physically active individuals had a lower association between PiB-PET Aβ burden with age compared to inactive individuals (Okonkwo et al., 2014). Aβ can also be measured within cerebrospinal fluid (CSF), with lower CSF Aβ associated with higher PiB-PET Aβ measures (Fagan et al., 2006). This negative association was supported by a meta-analysis of 131 studies (Olsson et al., 2016), and is thought to be due to higher levels of Aβ aggregated in plaques in the brain leaving less Aβ available to be secreted to the CSF. Cross-sectional evidence suggests that physical activity is positively associated with CSF Aβ (Law et al., 2018), consistent with physical activity being associated with reduced brain Aβ. Blood plasma Aβ mirrors the profile seen in CSF (Blennow and Zetterberg, 2018), with plasma Aβ being lower in individuals with high PiB-PET Aβ (Ovod et al., 2017). Finally, erythrocytes (red blood cells) can be used to measure Aβ (Lan et al., 2015). While less research has been conducted in this area, erythrocyte Aβ accumulation increases with age, and the profile does not follow the reversed pattern seen in CSF and blood plasma (Kiko et al., 2012). Less is known about the association between physical activity and tau. A recent review concluded that evidence for an association between physical activity and reduced tau (and brain Aβ) is robust in mice, with longitudinal studies potentially supporting a causative effect, but that more research is needed to confirm the association in humans (Brown et al., 2019).

Physical activity also appears to have a positive effect on brain structure. Higher levels of physical activity have been associated with larger grey matter (GM) volumes, particularly in frontal and temporal regions (Bugg and Head, 2011). A randomised controlled trial revealed increased frontal cortical thickness in participants who engaged in aerobic exercise, supporting a causal relationship (Stern et al., 2019). White matter (WM) structure, another key factor in maintaining brain health, has also been positively associated with being physically active (Marks et al., 2007). Evidence again suggests a causal relationship, with a 6-month randomised aerobic exercise intervention resulting in increased WM volume (Colcombe et al., 2006).

An aspect of brain structure which is less easy to interpret is WM integrity, which is inferred from measures of water diffusion in brain tissue. When diffusion is constrained along an axis, it is said to be anisotropic, and is thought to reflect the structure of axons. While lower mean diffusivity (MD) and higher fractional anisotropy (FA) suggest more constrained diffusion of water and therefore better WM integrity, crossing neural fibres mean that this conclusion must be made with caution. Diffusion can appear more isotropic as axons intersect in complex architectural regions despite high structural integrity (Pierpaoli and Basser, 1996; Madden et al., 2009). It is therefore suggested that these measures are not automatically interpreted as indicating WM integrity (Jones et al., 2013). Cerebrovascular health is another important factor for maintaining cognitive ability. The presence of white matter hyperintensities (WMH) observed by MRI are thought to indicate poorer cerebrovascular health (Wardlaw et al., 2015), and physical fitness has been associated with reduced WMH (Ritchie et al., 2017).

In terms of brain activation, physical activity is associated with the strength of task-related neural activation. A meta-analysis of 20 studies which investigated a range of cognitive tasks reported that physical activity was associated with parietal lobe activation, specifically in the precuneus (Yu et al., 2021), which is often affected in the early stages of AD (Jacobs et al., 2012). Communication between brain regions may also benefit from physical activity. During an executive control task, physically active individuals showed greater functional connectivity compared to physically inactive participants (Kamijo et al., 2011). Though functional connectivity is generally considered beneficial, strong synchronicity between two regions could be indicative of a deficit, given evidence of oscillatory hypersynchrony in AD mice (Vico Varela et al., 2019). This must therefore be considered when interpreting the association between physical activity and functional connectivity.

### Apolipoprotein E and Brain Health

Though physical activity is a promising target for promoting brain health, it is important to know whether it benefits everyone equally. Research has focused on a number of potential moderators of physical activity-brain health associations, including genetic factors such as *APOE* genotype. Apolipoprotein E is a protein involved in cholesterol transportation (Mahley, 1988; Bennet et al., 2007). The gene (*APOE*) which codes for this protein comes in three different versions—or alleles—known as e2, e3, and e4. The e4 allele is estimated to have a frequency of 14.4% in the UK (Corbo and Scacchi, 1999) and is associated with increased risk of AD (Corder et al., 1993), vascular dementia (VD) (Chuang et al., 2010), and stroke (Khan et al., 2013). Around 95% of AD cases are sporadic late onset, and e4 possession confers the strongest known genetic risk for late onset AD (Rocchi et al., 2003). Estimates of the variance in late onset AD diagnosis explained by *APOE* range from 6 to 13% (Ridge et al., 2013, 2016).

Possession of the e4 allele is also associated with cognitive decline within what might be considered “typical” age-related changes, though some of those “typical” changes may actually result from prodromal stages of dementia, with decline identified up to 6 years prior to diagnosis (Wilson et al., 2011). Whatever the mechanism, a meta-analysis demonstrated impaired cognitive ability in middle-aged e4 carriers compared to non-carriers, suggesting a cognitive phenotype prior to clinical diagnosis (Wisdom et al., 2011). *APOE* e4 possession has been associated with poorer outcomes in lipid profile (Leoni et al., 2010; Ferguson et al., 2020), Aβ burden (Liu et al., 2015), GM volume (Wishart et al., 2006), WM integrity (Persson et al., 2006; Operto et al., 2018), cerebrovascular health (Rojas et al., 2018; Lyall et al., 2019), task-related neural activation (Bondi et al., 2005) and functional connectivity (Canuet et al., 2012), i.e., the factors that appear to benefit from engagement in physical activity described earlier.

### APOE Moderation of the Association Between Physical Activity and Brain Health

Evidence suggests that the benefit of physical activity for brain health may differ by *APOE* status, however, findings have been inconsistent. For example, studies have shown cognitive ability to be associated with physical activity in either e4 carriers (Pizzie et al., 2014) or e4 non-carriers only (Obisesan et al., 2012). Other studies have shown an association between physical activity and cognitive ability in both e4 carriers and non-carriers (Sabia et al., 2010; Rodriguez et al., 2018). A recent systematic review investigating the association between physical activity, dementia risk and brain health suggested that e4 carriers might show a stronger association between physical activity and amyloid burden, and that in some cases only e4 carriers, and in others both carriers and non-carriers, showed an association between physical activity and functional neuroimaging outcomes (de Frutos-Lucas et al., 2020c). The authors concluded that while there was some evidence of moderation by *APOE*, the overall picture was inconclusive.

In the present review, we considered the moderating effect of *APOE* on the association between physical activity and a broader range of outcomes including lipid profile (LDL, HDL, TC), AD pathology (Aβ and tau), brain structure (GM volume, WM volume, WM integrity and cerebrovascular health) and brain activation (task-related activation, resting-state activation, resting-state functional connectivity). In addition to narrative syntheses, we conducted additional meta-analyses where possible to empirically investigate the nature and extent of any *APOE* moderation.

## Methods

This systematic review and meta-analysis is reported in accordance with the Preferred Reporting Items for Systematic Reviews and Meta-Analyses (PRISMA). A protocol (CRD42020164913) for this review was registered with PROSPERO and the record can be accessed online: https://www.crd.york.ac.uk/prospero/display_record.php?RecordID=164913.

### Search Strategy

Initial searches were conducted in February 2020 for peer reviewed studies written in English in PubMed, PsycINFO, Web of Science and SCOPUS. Search strings included terms relating to physical activity (e.g., “physical activity” or “exercise”), *APOE* (e.g., “apolipoprotein E” or “e4”), and the outcomes (e.g., “amyloid” or “grey matter”) (see Supplementary Table 1 for full search terms). A second search was carried out to include all studies published up to 31st December 2020. To yield additional studies, reference lists of review papers returned from the searches were examined along with searches of the lead author's records.

### Inclusion Criteria

Cross-sectional, longitudinal and intervention studies with adults aged 18 or over were eligible for inclusion. Studies including healthy participants or those with mild cognitive impairment were included, but studies which only assessed participants diagnosed with dementia were excluded. Studies were required to examine the association between total physical activity or physical fitness and one of the outcomes with a comparison of the association by *APOE* status. This could be through a statistical assessment of a physical activity by *APOE* interaction, or by stratified analyses for e4 carriers and non-carriers. Carriers included participants carrying either one e4 allele (heterozygotes) or two e4 alleles (homozygotes).

### Selection Process

Search results were combined in EndNote and duplicates removed. Titles and abstracts were screened by one reviewer (AP). Full text screening was carried out independently by two reviewers (AP and CM) with any discrepancies discussed until consensus was achieved.

### Data Extraction

Study characteristics extracted included study design, population, outcome(s), physical activity measure and *APOE* genotype. If cross-sectional data and longitudinal change were reported in the same paper, longitudinal outcomes were extracted. Data extracted included main effects of physical activity and *APOE*, and the interaction term if applicable. Associations between physical activity and the outcome were extracted for e4 carriers and non-carriers separately. Where relevant data were not reported, an email request was sent to the authors. One reminder email was sent after 3 weeks if there had been no response.

### Analysis

Narrative syntheses consisted of a discussion of the association between physical activity and each outcome, and whether the association differed depending on *APOE* genotype. For meta-analyses to be possible, at least 5 studies were required. As TC levels can be misleading, they were not deemed suitable for meta-analysis, and as high LDL represents a negative outcome and HDL represents a positive outcome, they were assessed in two separate meta-analyses. Similarly, interpretation of WM integrity is ambiguous where there are crossing neural fibres, so only a narrative synthesis was deemed possible.

When meta-analysis was possible, effect sizes of associations between physical activity and the outcome from each study were included separately for e4 carriers and non-carriers. A subgroup analysis was used to determine whether any association between physical activity and the outcomes differed by *APOE* status.

Where an outcome was analysed with different measurements or techniques, all effect sizes were included in the meta-analysis. To account for the resulting dependency from multiple effect sizes being obtained from the same sample, a multilevel model was used. Simulations suggest that multilevel models provide appropriate estimates of mean effects and confidence intervals (Van den Noortgate et al., 2014), and are considered superior to alternatives such as computing an average or selecting one effect size from each study as these do not utilise the available data (Cheung, 2019). Analyses were conducted in R Core Team (2020) using the metafor v2.4-0 package (Viechtbauer, 2010) with effect sizes nested within their respective study. Comparisons were made between the full multilevel model and a model with the study level held constant at zero to determine whether the multilevel model provided a better fit. Where the Bayesian Information Criterion (BIC) and the Akaike Information Criterion (AIC) were significantly lower in the multilevel model, the multilevel meta-analysis was used (Assink and Wibbelink, 2016), but where the full model did not provide a better fit, the standard meta-analysis was retained.

Due to the expected heterogeneity among study designs and outcomes, random effects models were used. In contrast to a fixed effect model which assumes one true effect size, a random effects model assumes a distribution of true effect sizes. Heterogeneity was assessed with the I^2^ statistic, which indicates the extent to which studies differ over and above random sampling error. Where heterogeneity was high, study characteristics and forest plots were examined to identify differences which could explain this heterogeneity. Where appropriate, *post-hoc* sensitivity analyses were carried out with potential sources of heterogeneity removed from meta-analyses to identify where studies differed.

The metric used to estimate summary effects was Pearson's *r*. If this was not reported, the Campbell Collaboration effect size calculator (https://campbellcollaboration.org/research-resources/effect-size-calculator.html) was used to convert *r* from either (1) standardised or unstandardised regression coefficient and sample size; (2) means, standard deviations and sample sizes (where there were more than two physical activity groups, the most active and the least active were used); (3) *t*-test *t*-value and sample sizes; or (4) *t*-test *p*-value and sample sizes. Where rho was reported, this was used instead of Pearson's *r* as this was preferable to omitting the data.

Where necessary, the sign of a correlation was reversed to ensure that associations between physical activity and outcomes were consistent. For example, effect sizes for the associations between physical activity and CSF Aβ and blood plasma Aβ were reversed so that positive values represented greater brain Aβ burden. One study reversed the PiB PET Aβ sign so that larger positive values corresponded to lower Aβ burden (Vemuri et al., 2016), reported as a positive correlation though interpreted as a higher level of physical activity being associated with less Aβ. In the current review, that correlation was reported consistent with effect sizes from other studies considering PiB PET and erythrocytes, where a negative correlation indicated that brain Aβ burden was lower in those reporting higher physical activity. For functional brain outcomes, shorter latencies resulted in a negative correlation with physical activity, and these were reversed so that a positive correlation indicated a better outcome associated with physical activity.

Some studies which reported a non-significant physical activity by *APOE* interaction did not present the stratified data. Where these data could not be obtained after email request, the missing data were imputed. A technique common in meta-analyses where non-significant odds ratios are unavailable is to set the odds ratio to 1. As the aim of the analysis was to use a subgroup analysis to assess whether the association between physical activity and the outcome differed by *APOE* status, where the stratified effects for e4 carriers and non-carriers were not available separately, the Pearson's *r* main effect of physical activity for e4 carriers and non-carriers combined was used for both e4 carriers and non-carriers individually, effectively setting the difference across *APOE* to 0. If the physical activity main effect was also not reported, this was set to 0 for both e4 carriers and non-carriers. Where there was a significant physical activity by *APOE* interaction but one of the stratified analyses was non-significant and not reported, this was set to 0. The alpha level for significance tests for all analyses was *p* = 0.05 or a 95% confidence interval.

### Publication Bias

Contour enhanced funnel plots were generated using the metafor v2.4-0 package (Viechtbauer, 2010) in R Studio and used to visually investigate publication bias. When multiple outcomes from one study were included in the analysis, all effect sizes were included in the funnel plot grouped by symbol to aid judgement. Subgroups of effect sizes for e4 carriers and non-carriers were colour coded so that a judgement of any bias across *APOE* genotype could be made.

### Study Quality

Study quality was assessed using the National Heart, Lung and Blood Institute's Quality Assessment Tool for Observational Cohort and Cross-Sectional Studies. The tool includes 14 items designed to assess study quality, assessing, for example, how participants were selected and compared; whether exposures and outcomes were valid and reliable; and whether potential confounds had been accounted for. An overall judgement determined whether each study was good, fair or poor. The assessment tool does not specify a scoring system for determining overall quality but is designed to help the user focus on key aspects of study quality from which an overall judgement can be made. Though all items were used to form an overall judgement, items 6, 7, 8, and 14 were critical in judging a study as good or bad. These items focused on the possible variance in the physical activity measures, whether those were taken prior to the outcome measure with sufficient time for an effect to be seen, and whether key confounding variables were accounted for. Assessment was carried out independently by two reviewers (AP and CM) with any discrepancies discussed until consensus was achieved.

## Results

### Study Selection

**Figure 1:**
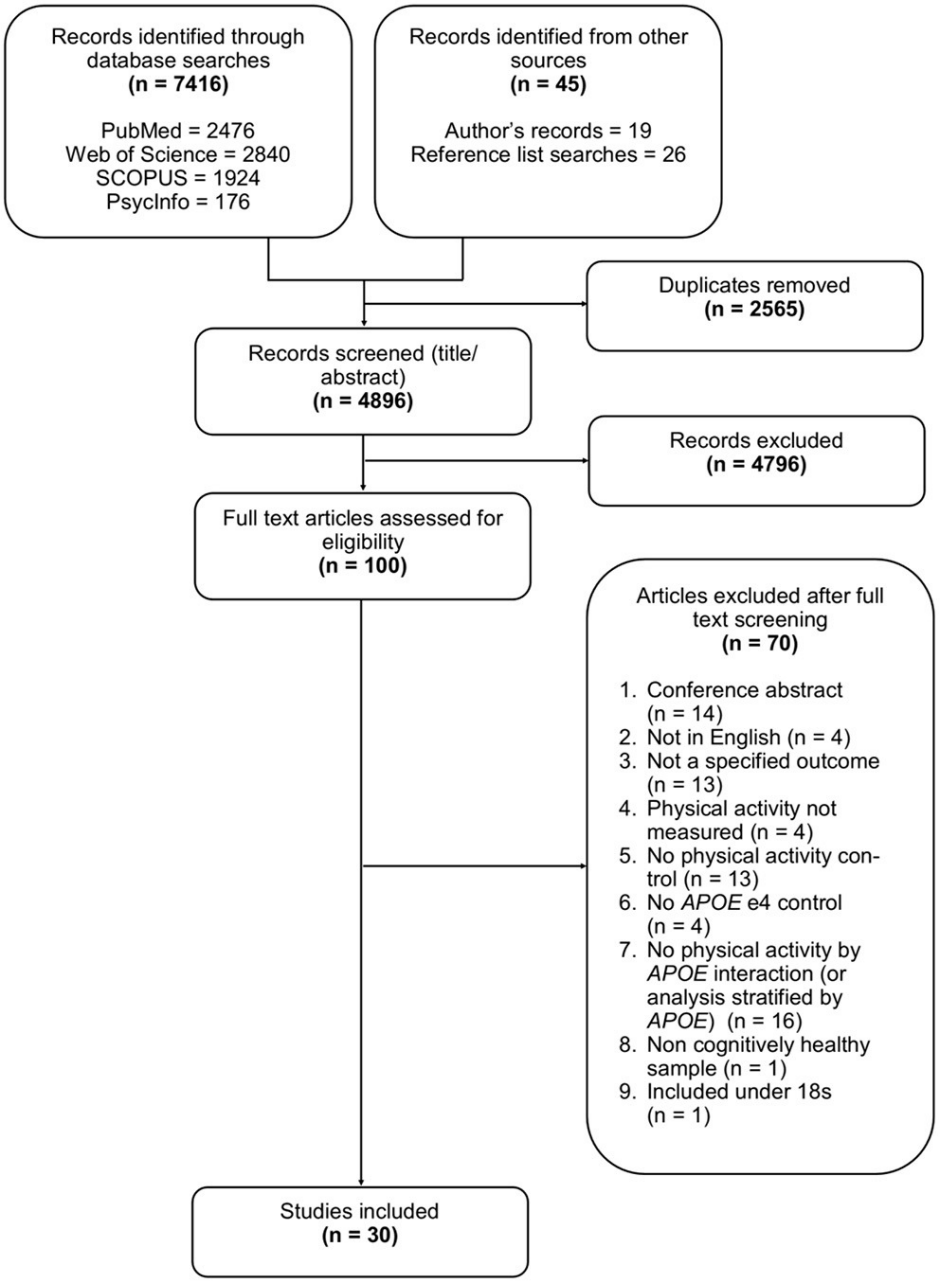
Flow diagram detailing the selection process for study inclusion.

After reviewing the titles and abstracts of 4,896 studies, 100 underwent full text review, with 30 selected for inclusion, some of which contributed to multiple outcomes. Of the 30 studies, eight assessed lipid profile, eight assessed AD pathology, six assessed brain structure, and nine assessed brain activation. Full details of the search results and selection process are illustrated in Figure 1, and study characteristics are given in Table 1.

**Table 1:** Study characteristics for all included studies assessing *APOE* differences in the association between physical activity and all outcomes.

**Study**	**Country**	**Population**	**Total sample size**	**Mean age (SD) total sample**	**Age range total sample**	**Study design**	**Outcome(s)**	**PA assessment**	**PA variable continuous or categorical**	**PA measure(s)/categories (number of participants per condition)**	***APOE* groups (number of participants per genotype)**
Bernstein et al. (2002)	Switzerland	Healthy general population	1,708	NR	35–74	Cross sectional	HDL; LDL; TC	Questionnaire (Physical Activity-Frequency Questionnaire)	Continuous	% of PA at high intensity	e4+ (*n* = 320) e3e3 (*n* = 1170) e2e4 excluded
Boer et al. (1997)	France	Healthy sample from the European Atherosclerosis Research Study	1474	NR	18–26	Cross sectional or retrospective cohort (time of PA measure NR)	TC	Questionnaire	Continuous	PA measure not reported in detail	e4+ (*n* = 381) e3e3 (*n* = 915) e2e4 excluded
Boer et al. (1998)	Netherlands	Healthy sample from the Cardiovascular Disease Risk Factor Monitoring Project	294	45.6 (10.8)	NR	Cross sectional or retrospective cohort (time of PA measure NR)	Lipid risk profile	Questionnaire	Categorical	Active (*n* = 192) Inactive (*n* = 102)	e4+ (*n* = 67) e3e3 (*n* = 189) e2e4 excluded
Boots et al. (2015)	USA	Subsample from the Wisconsin Registry for Alzheimer's Prevention Longitudinal Cohort	315	58.6 (6.3)	40–65	Cross sectional	GM volume; WMH	Cardiorespiratory fitness	Continuous	Cardiorespiratory fitness	e4+ (*n* = 122) e4– (*n* = 193)
Brown et al. (2013)	Australia	Healthy sample from the Australian Imaging, Biomarkers and Lifestyle Study of Ageing	Blood plasma analysis: 546 PiB PET analysis: 116	69.6 (6.8)	60–95	Cross sectional	Aβ_42/40_ (blood plasma [INNO-BIA and ELISA assays]); Aβ (PiB PET)	Questionnaire (International Physical Activity Questionnaire)	Categorical	Blood plasma analysis: T3 (most active) (*n* = 182) T2 (*n* = 182) T1 (least active) (*n* = 182) PiB PET analysis: T3 (most active) (*n* = 38) T2 (*n* = 39) T1 (least active) (*n* = 39)	Blood plasma analysis: e4+ (*n* = 148) e4– (*n* = 398) PiB PET analysis: e4+ (*n* = 55) e4– (*n* = 61)
Corella et al. (2001)	Spain	Healthy sample from the Population Survey on Cardiovascular Risk Factors	819	36.7 (10.3)^a^	18–66	Cross sectional	HDL; LDL	Questionnaire	Categorical	Active (*n*= 253) Sedentary (*n* = 511)	e4+ (*n* = 119) e3e3 (*n* = 687) e2e4 excluded^b^
de Frutos-Lucas et al. (2018)	Spain	Healthy community dwelling sample	100	71.8 (4.3)	65+^c^	Cross sectional	Resting-state individual alpha peak frequency (MEG)	Questionnaire (International Physical Activity Questionnaire)	Categorical	High PA (*n* = 16) Moderate PA (*n* = 60) Low PA (*n* = 24)	e3e4 (*n* = 20) e3e3 (*n* = 80)
de Frutos-Lucas et al. (2020a)	Spain	Healthy participants recruited from local hospitals (MMSE ≥26)	107	60.5 (8.1)	50–82	Cross sectional	Resting-state functional connectivity between temporal lobe and whole brain or specific seed regions (MEG)	Accelerometer worn over 7 days	Continuous	Total PA	e3e4 (*n* = 33) e3e3 (*n* = 74)
de Frutos-Lucas et al. (2020b)	Spain	Healthy sample recruited from local hospitals (MMSE ≥26)	113	59.9 (7.5)	48–82	Cross sectional	Resting-state alpha band power (MEG)	Accelerometer worn over 7 days	Continuous	Total PA	e3e4 (*n* = 36) e3e3 (*n* = 77)
de Souto Barreto et al. (2015)	France	Healthy sample reporting memory complaints which affect one or more aspects of daily living (clinical dementia rating <1)	268	74.7 (4.2)	70+^c^	Cross sectional	Aβ (Florbetapir PET)	Questionnaire	Continuous	Total PA	e4+ (*n* = 65) e4– (*n* = 169)
Deeny et al. (2008)	USA	Healthy (screened with Cambridge Cognitive Exam) sample recruited through newspaper advertisements, local running events and campus staff	23	59.5 (5.1)	50–70	Cross sectional	Task-related cortical activation (MEG M170 amplitude and latency)	Questionnaire (Yale Physical Activity Survey)	Categorical	High active (*n* = 14) Low active (*n* = 9)	e4+ (*n* = 9) e4– (*n* = 14) e2e4 excluded
Gu et al. (2020)	USA	Healthy sample selected from the Washington/Hamilton Heights-Inwood Columbia Aging Project	1,389	77.2 (6.4)	65+^c^	Cross sectional	GM volume; WM volume; WMH	Questionnaire (Godin Leisure Time Exercise)	Categorical	High active (*n* = 357) Moderately active (*n* = 382) Low active (*n* = 346) Inactive (*n* = 304)	e4+ (*n* = 386) e4– (*n* = 1,003)
Gustavsson et al. (2012)	Sweden	Healthy participants from two cohorts: Interplay between genetic susceptibility and environmental factors for the risk of chronic diseases in West Sweden; Stockholm Heart Epidemiology Program	6,389	55.8 (11.1)^a^	NR	Retrospective cohort	LDL	Questionnaire	Categorical	Active (*n* = 4,933) Inactive (*n* = 1,456)	e4e4 (*n* = 171) e3e4 (*n* = 1591) e3e3 (*n* = 3,733)
Head et al. (2012)	USA	Cognitively healthy sample (classified with the Washington University Clinical Dementia Rating)	CSF analysis: 165 PiB PET analysis: 163	CSF analysis: 66.5 (9.6)^a^ PiB PET analysis: 67 (10)^a^	45-88	Retrospective cohort	Aβ_42_ (CSF); Aβ (PiB PET)	Questionnaire	Categorical	CSF analysis: High exercise (*n* = 35) Low exercise (*n* = 130) PiB PET analysis: High exercise (*n* = 38) Low exercise (*n* = 125)	CSF analysis: e4+ (*n* = 56) e4– (*n* = 109) PiB PET analysis: e4+ (*n* = 52) e4– (*n* = 111)
Honea et al. (2009)	USA	Cognitively healthy sample (Clinical Dementia Rating = 0) from the University of Kansas Brain Aging Project	56	73.3 (6.2)	65+^c^	Cross sectional	GM volume; WM volume	Cardiorespiratory fitness	Continuous	${\text{VO}}_{2}^{\text{peak}}$ (mL/kg/min)	e4+ (*n* = 18) e4– (*n* = 29)
Jeon et al. (2020)	South Korea	Dementia free sample (215 cognitively healthy, 72 MCI) from the Korean Brain Aging Study for the Early Diagnosis and Prediction of Alzheimer's Disease	287	71.9 (6.6)	55–90	Retrospective cohort	Aβ (PiB PET)	Questionnaire (Lifetime Total Physical Activity Questionnaire)	Continuous	Midlife (40–55 years) leisure activity	e4+ (*n* = 66) e4– (*n* = 221)
Kerestes et al. (2015)	USA	Subjective memory complainers who performed within normal range on a cognitive battery from the Consortium to Establish a Registry for Alzheimer's Disease	80	72.6 (5.7)^a^	NR	Cross sectional	Resting-state functional connectivity in the posterior cingulate cortex	Pedometer	Continuous	Steps per week	e4+ (*n* = 22) e4– (*n* = 58)
Liang et al. (2010)	USA	Cognitively healthy sample (classified with the Washington University Clinical Dementia Rating)	CSF analysis: 56 PiB PET analysis: 54	CSF analysis: 66.46 (8.7)^a^ PiB PET analysis: 70.4 (10)^a^	55–88	Retrospective cohort	Aβ_42_ (CSF); Aβ (PiB PET) Tau pTau	Questionnaire	Categorical	CSF analysis: High exercise (*n* = 10) Low exercise (*n* = 46) PiB PET analysis: High exercise (*n* = 11) Low exercise (*n* = 43)	CSF analysis: e4+ (*n* = 21) e4– (*n* = 35) PiB PET analysis: e4+ (*n* = 16) e4– (*n* = 38)
Piccarducci et al. (2019)	Italy	Healthy sample recruited from the University of Pisa	42	39.7 (13.2)^a^	20–70	Cross sectional	Aβ (erythrocytes)	Questionnaire (Borg scale of Perceived Exertion)	Categorical	Active (*n* = 21) Non-active (*n* = 21)	e4+ (*n* = 16) e4– (*n* = 26)
Pisciotta et al. (2003)	Italy	Healthy sample	200	50.9 (7.1)^a^	40+^c^	Cross sectional	HDL; LDL; TC	Cycling engagement	Categorical	Active (*n* = 100) Sedentary (*n* = 100)	e4+ (*n* = 27) e3e3 (*n* = 144) no e2e4
Schmitz et al. (2001)	USA	Healthy sample from the Coronary Artery Risk Development in Young Adults study	3,629	25 (0.08)^a^	18–30	Prospective cohort	HDL; LDL	Physical fitness change	Categorical	Increase (*n* = 283) Maintain (*n* = 825) Decrease (*n* = 1139)	e4+ (*n* = 1001) e3e3 (*n* = 2033) e2+ (*n* = 592) e2e4 excluded
Smith et al. (2011)	USA	Healthy sample recruited from newspaper advertisements	68	72.8 (4.8)^a^	65–85	Cross sectional	Task-related brain activation (fMRI BOLD)	Questionnaire (Stanford Brief Activity Survey)	Categorical	High PA (*n* = 34) Low PA (*n* = 34)	e4+ (*n*= 34) e4– (*n* = 34)
Smith et al. (2014)	USA	Healthy sample recruited from newspaper advertisements	97	72.9 (4.8)^a^	65–89	Prospective cohort	GM change; WM change	Questionnaire (Stanford Brief Activity Survey)	Categorical	High PA (*n* = 46) Low PA (*n* = 51)	e4+ (*n* = 39) e4– (*n* = 58)
Smith et al. (2016)	USA	Healthy sample recruited from newspaper advertisements	88	74.1 (4.6)^a^	65–89	Cross sectional	WM integrity	Questionnaire (Stanford Brief Activity Survey)	Categorical	High PA (*n* = 41) Low PA (*n* = 47)	e4+ (*n* = 34) e4– (*n* = 54)
St-Amand et al. (1999)	Canada	Healthy sample recruited through media advertisements	129	35.9 (3.91)^a^	25–48	Cross sectional	HDL; LDL; TC	Cardiorespiratory fitness	Continuous	${\text{VO}}_{2}^{\text{peak}}$ (mL/kg/min)	e4+ (*n* = 29) e3e3 (*n* = 63) e2e4 excluded
Stojanovic et al. (2020)	USA	Cognitively healthy sample (classified with the Washington University Clinical Dementia Rating)	CSF analysis: 95 PiB PET analysis: 181	CSF analysis: 62.7 (7.7)^a^ PiB PET analysis: 67.3 (9.3)^a^	55–88	Retrospective cohort^d^	Aβ_42_ (CSF); Aβ (PiB PET) Tau pTau	Questionnaire	Categorical	CSF analysis: High exercise (*n* = 33) Low exercise (*n* = 62) PiB PET analysis: High exercise (*n* = 59) Low exercise (*n* = 122)	CSF analysis: e4+ (*n* = 34) e4– (*n* = 61) PiB PET analysis: e4+ (*n* = 56) e4– (*n* = 125)
Tsai et al. (2019)	Taiwan	Healthy sample with a family history of Alzheimer's disease	32	53.6 (7.8)^a^	30–70	Cross sectional	Task-related alpha band oscillations EEG	${\text{VO}}_{2}^{\text{max}}$ Estimate from the Rockport Fitness Walking Test	Continuous	${\text{VO}}_{2}^{\text{max}}$ estimate	e4+ (*n* = 16) e4– (*n* = 16) no e2e4
Tsai et al. (2021)	Taiwan	Healthy sample with a first-degree family history of Alzheimer's disease (at least one parent with Alzheimer's disease)	44	58.5 (6.5)^a^	38–73	Cross sectional	Aβ_42_ (blood plasma); Task-related EEG amplitudes	${\text{VO}}_{2}^{\text{max}}$ Estimate from the Rockport Fitness Walking Test	Continuous	${\text{VO}}_{2}^{\text{max}}$ estimate	e4+ (*n* = 22) e4– (*n* = 22)
Vemuri et al. (2016)	USA	Dementia free sample (340 cognitively healthy, 53 MCI) recruited from the Mayo Clinic Study of Aging	393	78.6 (5)^a^	70+^c^	Prospective cohort^e^	Aβ (PiB PET) GM volume	Questionnaire	Continuous	Midlife (50–65 years) PA	e4+ (*n* = 109) e4– (*n* = 284)
Zlatar et al. (2014)	USA	Healthy community dwelling sample from an ongoing research study at the University of California	33	69 (8.5)^a^	52–81	Cross sectional	Resting-state hippocampal blood flow (ASL MRI)	Accelerometer worn over 7 days	Continuous	Daily hours sedentary Total PA/hour	e4+ (*n* = 9) e4– (*n* = 24) no e2e4

*Aβ, Amyloid beta; APOE, Apolipoprotein E; ASL, Arterial spin labelling; BOLD, Blood oxygen level dependent; CSF, Cerebrospinal fluid; e4–, No APOE e4 alleles; e4+, Carrier of one or two APOE e4 alleles (includes e2e4 genotype unless stated otherwise); EEG, Electroencephalogram; fMRI, Functional magnetic resonance imaging; GM, Grey matter; HDL, High density lipoprotein; LDL, Low density lipoprotein; MCI, Mild cognitive impairment; MEG, Magnetoencephalogram; NR, Not reported; PA, Physical activity; PiB, Pittsburgh compound B; PET, Positron emission tomography; TC, Total cholesterol; WM, White matter; WMH, White matter hyperintensities*.

a *Calculated from mean age and SD reported separately by genotype*.

b *Genotype frequencies for sample (n = 909) selected from broader population survey prior to selection of study sample. Genotype frequencies not reported for study sample*.

c *No upper age limit reported*.

d *Study looked at prospective outcomes but follow up sample contained participants with dementia diagnosis, so only baseline data were used in this review*.

e *Study looked at prospective outcomes but the data provided for this review were from baseline*.

### Lipid Profile

Of the eight studies assessing lipid profile, six assessed LDL, five assessed HDL, and four assessed TC. One study did not assess lipoprotein levels individually, instead calculating an overall lipid risk score as a dichotomous outcome (see Table 2 for lipid data).

**Table 2:** Associations between physical activity and lipid profile.

**Outcome**	**Study**	**PA measurement**	**Outcome measure**	**Gender**	**PA association with outcome**	***APOE* association with outcome**	**PA x *APOE* interaction association with outcome**	**Association of PA with outcome according to** ***APOE*** **status**	
								**e4–**	**e4+**
LDL	Bernstein et al. (2002)	% of PA at high intensity (activities which exert ≥ 4 times the basal metabolic rate)^d^	LDL cholesterol (mmol/L)	Male	Tertile group means = NR *t* = NR, *p* > 0 .05	e3: 3.81 (0.04) e4: 4.01 (0.06) *t* = NR, *p* < 0.05^a^	*B* = NR, *p* = 0.16	*B* = −0.0009, *p* = 0.75	*B* = 0.0085, *p* = 0.15
				Female	Tertile group means = NR *t* = NR, *p* > 0.05	e3: 3.81 (0.04) e4: 4.01 (0.06) *t* = NR, *p* < 0.05^a^	*B* = NR, *p* = 0.19	*B* = −0.0013, *p* = 0.78	*B* = −0.0133, *p* = 0.11
	Corella et al. (2001)	Active: engaging in at least one sport per week (*n* = 253) Sedentary: no exercise (*n* = 511)	LDL cholesterol (mg/dL)	Male	*B* = 0.2, SE = 3.5, *p* = 0.944	*B* = 9.2, SE = 4.8, *p* = 0.054	*B* = NR, *p* = 0.704	Group means = NR *t* = NR, *p* = NR	Group means = NR *t* = NR, *p* = NR
				Female	*B* = 0.4, SE = 2.6, *p* = 0.889	*B* = 14.1, SE = 3.9, *p* < 0.001	*B* = NR, *p* = 0.882	Group means = NR *t* = NR, *p* = NR	Group means = NR *t* = NR, *p* = NR
	Gustavsson et al. (2012)	Active: moderate to hard exercise (*n* = 4,933) Inactive: very little PA Occasional walks but mainly sitting (*n* = 1,456)	LDL cholesterol (mmol/L)	Male and female	*B* = NR, *p* = NR	e3e3: 3.53 (SE = 0.02)^b^ e3e4: 3.73 (SE = 0.032)^b^ e4e4: 3.80 (SE = 0.10)^b^ *B* = NR, *p* = NR	*B* = NR, *p* > 0.05	Group means = NR *t* = NR, *p* = NR	Group means = NR *t* = NR, *p* = NR
	Pisciotta et al. (2003)	Active: 120–150 km/week road cycling (*n* = 100) Sedentary: non cycling age matched controls (*n* = 100)	LDL cholesterol (mmol/L)	Male	Active M = 3.37 (0.73) Sedentary M = 3.97 (0.93) *t* = NR, *p* < 0.001	Group means = NR	F = NR, *p* = NR	Active: 3.47 (0.66) Sedentary: 3.93 (0.84) *t* = NR, *p* > 0.01	Active: 3.37 (0.90)^c^ Sedentary: 4.71 (0.92)^c^ *t* = NR, *p* < 0.01
	Schmitz et al. (2001)	Seven-year change in seconds completed in a graded treadmill test: Increase (*n* = 283) Decrease (*n* = 1,139)	LDL cholesterol 7-year change (mg/dL)	Male	Increase: 2.95 (SE = 2.17) Decrease: 1.97 (SE = 0.93) *t* = NR, *p* > 0.05	e3: 2.28 (SE = 0.88) e4: 3.85 (SE = 1.32) *t* = NR, *p* > 0.05	F = 1.17, *p* = 0.32	Group means = NR *t* = NR, *p* = NR	Group means = NR *t* = NR, *p* = NR
				Female	Increase: −7.10 (SE = 1.53) Decrease: −4.67 (SE = 0.86) *t* = NR, *p* > 0.05	e3: −4.67 (SE = 0.76) e4: −3.13 (SE = 1.11) *t* = NR, *p* > 0.05	F = 0.91, *p* = 0.46	Group means = NR *t* = NR, *p* = NR	Group means = NR *t* = NR, *p* = NR
	St-Amand et al. (1999)	${\text{VO}}_{2}^{\text{peak}}$ (mL/kg/min)	LDL cholesterol (mmol/L)	Male	NR. Analysis stratified by *APOE*	e3: 3.51 (0.75) e4: 3.49 (0.83) *t* = NR, *p* > 0.05	NA. Analyses stratified by *APOE*	*r* = −0.10, *p* > 0.05	*r* = −0.21, *p* > 0.05
				Female	NR. Analysis stratified by *APOE*	e3: 3.48 (1.18) e4: 3.68 (0.88) *t* = NR, *p* > 0.05	NA. Analyses stratified by *APOE*	*r* = −0.39, *p* < 0.05	*r* = −0.04, *p* > 0.05
HDL	Bernstein et al. (2002)	% of PA at high intensity (activities which exert ≥ 4 times the basal metabolic rate)^d^	HDL cholesterol (mmol/L)	Male	Upper tertile: 1.25 Lower tertile: 1.19 *t* = NR, *p* < 0.004	e3: 1.34 (0.01) e4: 1.32 (0.02) *t* = NR, *p* > 0.05^a^	*B* = NR, *p* < 0.03	*B* = 0.0016, *p* = 0.09	*B* = 0.0066, *p* < 0.001
				Female	Tertile goup means = NR *t* = NR, *p* > 0.05	e3: 1.34 (0.01) e4: 1.32 (0.02) *t* = NR, *p* > 0.05^a^	*B* = NR, *p* = 0.21	*B* = 0.0012, *p* = 0.48	*B* = 0.0058, *p* = 0.07
	Corella et al. (2001)	Active: engaging in at least one sport per week (*n* = 253) Sedentary: no exercise (*n* = 511)	HDL cholesterol (mg/dL)	Male	*B* = −0.3, SE = 1.1, *p* = 0.792	*B* = −0.1, SE = 1.5, *p* = 0.953	*B* = NR, *p* = 0.001	Group means = NR *t* = NR, *p* = NR	Active: 48 (15) Sedentary: 38 (8) *t* = NR, *p* < 0.006
				Female	*B* = 0.1, SE = 1.0, *p* = 0.900	*B* = −0.8, SE = 1.5, *p* = 0.608	*B* = NR, *p* = 0.944	Group means = NR *t* = NR, *p* = NR	Group means = NR *t* = NR, *p* = NR
	Pisciotta et al. (2003)	Active: 120-150 Km/week of road cycling (*n* = 100) Sedentary: non cycling age matched controls (*n* = 100)	HDL cholesterol (mmol/L)	Male	Active: 1.58 (0.51) Sedentary: 1.34 (0.34) *t* = NR, *p* < 0.001	Group means = NR *t* = NR, *p* = NR	F = NR, *p* = NR	Active: 1.60 (0.51) Sedentary: 1.34 (0.33) *t* = NR, *p* = NR	Active: 1.43 (0.42)^c^ Sedentary: 1.23 (0.21)^c^ *t* = NR, *p* = NR
	Schmitz et al. (2001)	Seven-year change in seconds completed in a graded treadmill test: Increase (*n* = 283) Decrease (*n* = 1139)	HDL cholesterol seven-year change (mg/dL)	Male	Increase: −0.67 (SE = 0.79) Decrease: −2.86 (SE = 0.34) *t* = NR, *p* < 0.05	e3: −2.23 (SE = 0.32) e4:−2.41 (SE = 0.48) *t* = NR, *p* > 0.05	*F* = 0.99, *p* = 0.41	Group means = NR *t* = NR, *p* = NR	Group means = NR *t* = NR, *p* = NR
				Female	Increase: 0.53 (SE = 0.70) Decrease: −0.47 (SE = 0.39) *t* = NR, *p* > 0.05	e3: −0.08 (SE = 0.35) e4: −0.72 (SE = 0.5) *t* = NR, *p* > 0.05	*F* = 1.09, *p* = 0.36	Group means = NR *t* = NR, *p* = NR	Group means = NR *t* = NR, *p* = NR
	St-Amand et al. (1999)	${\text{VO}}_{2}^{\text{peak}}$ (mL/kg/min)	HDL cholesterol (mmol/L)	Male	NR. Analysis stratified by *APOE*	e3: 1.00 (0.24) e4: 1.00 (0.20) *t* = NR, *p* > 0.05	NA. Analyses stratified by *APOE*	*r* = 0.33, *p* < 0.05	*r* = 0.02, *p* > 0.05
				Female	NR. Analysis stratified by *APOE*	e3: 1.26 (0.27) e4: 1.09 (0.21) *t* = NR, *p* > 0.05	NA. Analyses stratified by *APOE*	*r* = 0.60, *p* < 0.001	*r* = 0.48, *p* < 0.05
TC	Bernstein et al. (2002)	% of PA at high intensity (activities which exert ≥4 times the basal metabolic rate)^d^	TC (mmol/L)	Male	Tertile group means = NR *t* = NR, *p* > 0.05	e3: 5.69 (0.04) e4: 5.91 (0.06) *t* = NR, *p* < 0.05^a^	*B* = NR, *p* = 0.26	*B* = 0.0000, *p* = 0.99	*B* = 0.0084, *p* = 0.21
				Female	Tertile group means = NR *t* = NR, *p* > 0.05	e3: 5.69 (0.04) e4: 5.91 (0.06) *t* = NR, *p* < 0.05^a^	*B* = NR, *p* = 0.53	*B* = −0.0082, *p* = 0.71	*B* = −0.0133, *p* = 0.36
	Boer et al. (1997)	PA measure not reported in detail	TC (mmol/L)	Male and female	NR. Analysis stratified by *APOE*	e3: 4.38 (0.03) e4: 4.62 (0.04) *t* = NR, *p* < 0.001	*B* = NR, *p* > 0.05	*r* = 0.01, *p* > 0.05	*r* = 0.05, *p* > 0.05
	Pisciotta et al. (2003)	Active: 120-150Km/week of road cycling (*n* = 100) Sedentary: non cycling age matched controls (*n* = 100)	TC (mmol/L)	Male	Active: 5.42 (0.80) Sedentary: 5.95 (1.05) *t* = NR, *p* < 0.001	Group means = NR *t* = NR, *p* = NR	*F* = NR, *p* = NR	Active: 5.55 (0.74) Sedentary: 5.90 (0.99) *t* = NR, *p* = NR	Active: 5.29 (0.87)^c^ Sedentary: 6.53 (1.06)^c^ *t* = NR, *p* = NR
	St-Amand et al. (1999)	${\text{VO}}_{2}^{\text{peak}}$ (mL/kg/min)	TC (mmol/L)	Male	NR. Analysis stratified by *APOE*	e3: 5.14 (0.84) e4: 5.03 (0.89) *t* = NR, *p* > 0.05	NA. Analyses stratified by *APOE*	*r* = −0.12, *p* > 0.05	*r* = 0.06, *p* > 0.05
				Female	NR. Analysis stratified by *APOE*	e3: 5.26 (1.24) e4: 5.26 (0.94) *t* = NR, *p* > .05	NA. Analyses stratified by *APOE*	*r* = −0.46, *p* < 0.01	*r* = 0.03, *p* > 0.05
Overall lipid risk profile	Boer et al. (1998)	Active: reported engaging in leisure time activity (*n* = 192), Inactive: reported no leisure time activity (*n* = 102)	High risk (TC above 85th percentile and HDL below 15th percentile) compared to median risk (TC and HDL within 42.5 to 57.5th percentile)	Male Female	Inactive: OR = 5.24 (1.30–21.1) Inactive: OR = 1.19 (0.54–2.66)	e4+: OR = 4.94 (1.06–23.1) e4+: OR = 1.29 (0.53–3.16)	*B* = NR, *p* > .05 *B* = NR, *p* > 0.05	OR = NR, *p* = NR OR = NR, *p* = NR	OR = NR, *p* = NR OR = NR, *p* = NR

*APOE, Apolipoprotein E; e4–, No APOE e4 alleles; e4+, Carrier of one or two APOE e4 alleles (includes e2e4 genotype unless stated otherwise); HDL, High density lipoprotein; LDL, Low density lipoprotein; NR, Not reported; NA, Not applicable; OR, Odds ratio; PA, Physical activity; TC, Total cholesterol. Additional data not included in the original publication are included in this review for Pisciotta et al. (2003)*.

a *Male and female combined*.

b *Calculated from mean LDL cholesterol reported separately for participants with and without coronary heart disease*.

c *Calculated from lipid concentrations reported separately for e3e4 and e4e4 participants*.

d *Continuous PA measurement split into tertiles for some analyses*.

#### Low Density Lipoproteins

**Figure 2:**
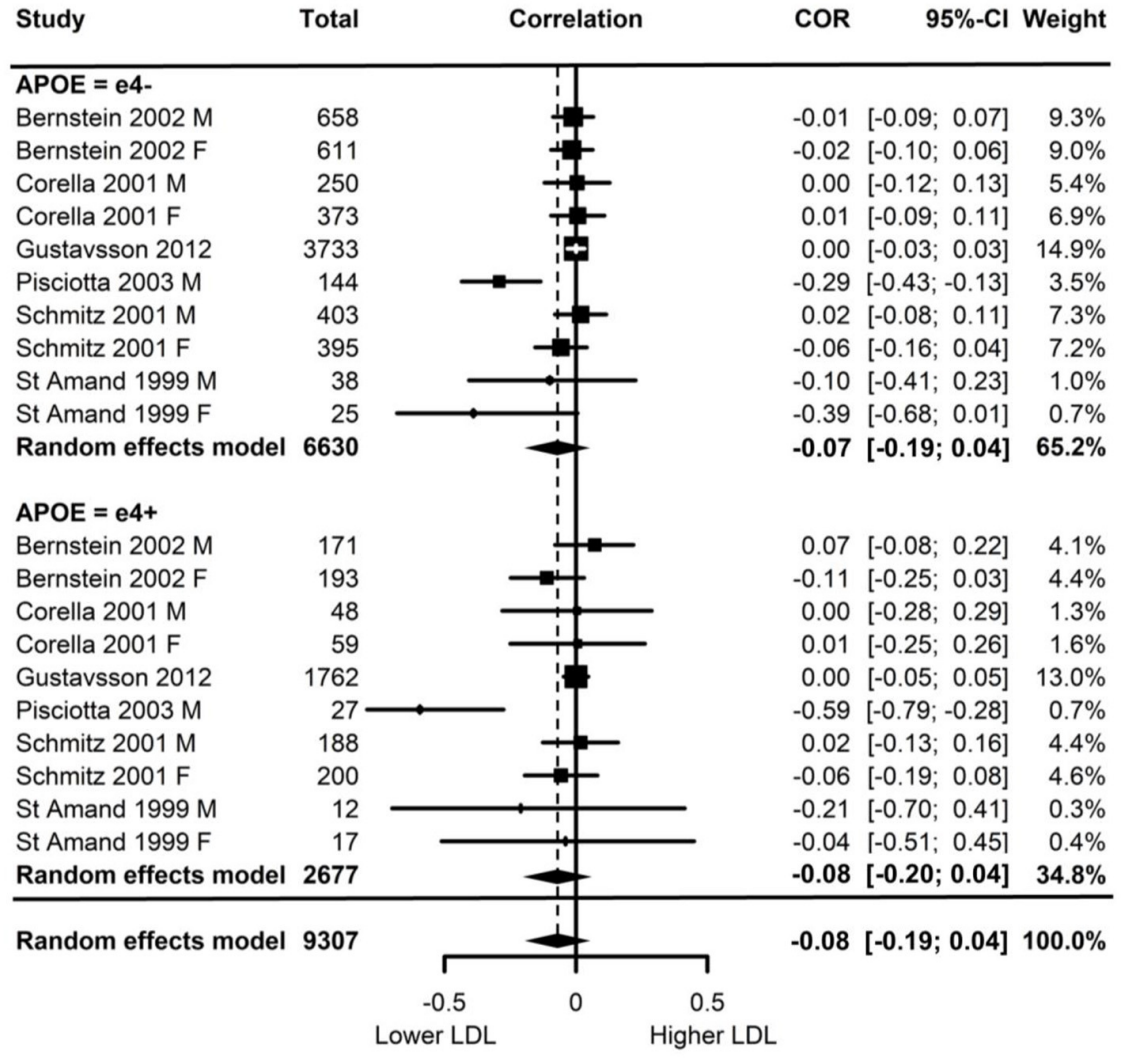
Forest plot indicating the association between physical activity and LDL with carrier (e4+) and non-carrier (e4–) subgroups. Subgroup moderation test indicated no significant difference between *APOE* groups (*p* = 0.84).

Of the six studies which assessed LDL, none showed moderation of the physical activity-LDL association by *APOE*. A meta-analysis was conducted with 10 effect sizes each for e4 carriers and non-carriers, five of which were substituted with the physical activity main effect from e4 carriers and non-carriers combined. Analysis of the AICs and BICs indicated that the multilevel model was a significantly better fit than the standard model (*p* = 0.014; see Supplementary Table 2 for model fit statistics). Physical activity was not significantly associated with LDL (*r* = −0.08, *p* = 0.17), and this was also the case for e4 carriers (*r* = 0.08, *p* = 0.18) and non-carriers (*r* = −0.07, *p* = 0.18) separately. The moderation test indicated that there was no significant difference between *APOE* subgroups [*F*_(1, 18)_ = 0.04, *p* = 0.84] (see Figure 2).

Visual inspection of the funnel plot (Supplementary Figure 1) indicated possible publication bias with smaller studies more likely to be published if demonstrating an association between physical activity and reduced LDL, however, this bias did not differ by *APOE* status.

Heterogeneity was high (I^2^ = 84.5%) and all of this variance was at the between cluster level (that is, effect sizes differed between studies but not within a study). *Post-hoc* investigation identified physical activity measurement, LDL measurement, and study design as possible sources of the between cluster heterogeneity. As the metrics used to quantify LDL can be directly converted, this was unlikely to be a source of heterogeneity. For study design, one study (Schmitz et al., 2001) assessed the association between physical activity and 7-year longitudinal change in LDL in contrast to the cross-sectional nature of the other studies. A sensitivity analysis with this longitudinal study removed again indicated high heterogeneity (I^2^ = 88.4%) with all of this variance was at the between cluster level.

#### High Density Lipoproteins

**Figure 3:**
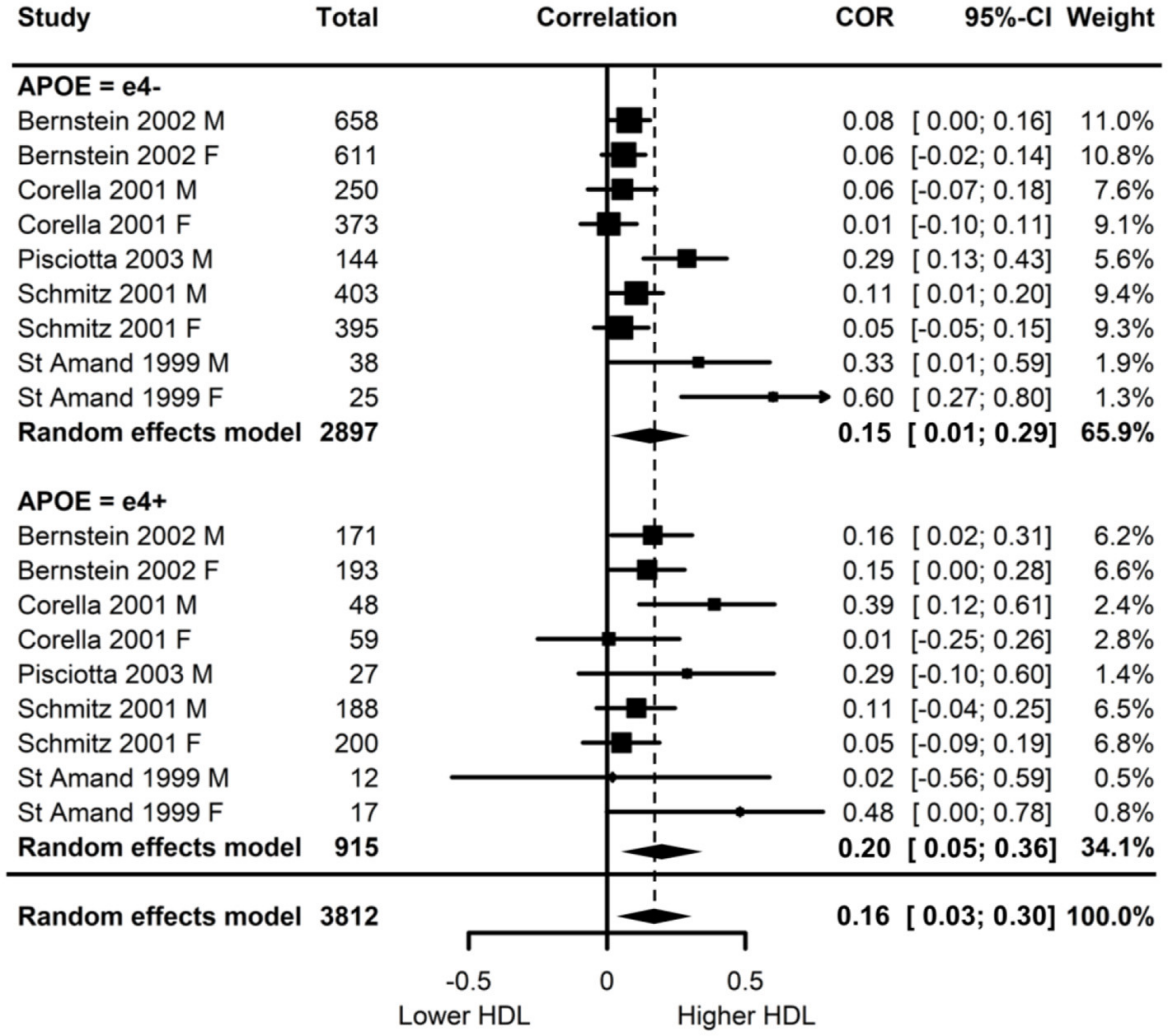
Forest plot indicating the association between physical activity and HDL with carrier (e4+) and non-carrier (e4–) subgroups. Subgroup moderation test indicated no significant difference between *APOE* groups (*p* = 0.10).

Of the five studies which assessed HDL, two provided evidence of *APOE* moderation of the physical activity-HDL association. A meta-analysis was conducted with nine effect sizes each for e4 carriers and non-carriers, three of which were substituted with the physical activity main effect from e4 carriers and non-carriers combined. AICs and BICs indicated that the multilevel model was a significantly better fit than the standard model (*p* = 0.03; see Supplementary Table 2 for model fit statistics). Physical activity was significantly associated with HDL (*r* = 0.16, *p* = 0.02), and this was also the case in the e4 carriers (*r* = 0.20, *p* = 0.01) and non-carriers (*r* = 0.15, *p* = 0.03) separately. The moderation test indicated that there was no significant difference between *APOE* subgroups [*F*_(1, 16)_ = 1.86, 0.19] (Figure 3).

Visual inspection of the funnel plot (Supplementary Figure 1) indicated possible publication bias with smaller studies more likely to be published if demonstrating an association between physical activity and increased HDL, however, this bias did not differ by *APOE* status.

Heterogeneity was high (I^2^ = 77.5%) and all at the between cluster level. *Post-hoc* investigation identified a similar pattern to the LDL analyses, with physical activity measurement, HDL measurement, and study design as possible sources of the between cluster heterogeneity. A sensitivity analysis with the longitudinal study removed made minimal difference, with high heterogeneity (I^2^ = 79.6%) again all at the between cluster level.

#### Total Cholesterol

Four studies assessed TC. One study did not report the physical activity by *APOE* interaction result or the stratified data (Pisciotta et al., 2003). Two studies reported the interaction result, both of which were not significant (Boer et al., 1997; Bernstein et al., 2002). The remaining study carried out stratified analyses across *APOE* and gender and only female non-carriers demonstrated a significant association between physical activity and TC (*r* = −0.46, *p* < 0.01; St-Amand et al., 1999).

#### Overall Lipid Risk Profile

One study assessed whether physical activity predicted a high-risk lipid profile (Boer et al., 1997). Participants with TC levels above the 85th percentile and HDL below the 15th percentile were compared to a medium risk profile consisting of participants with TC and HDL levels in the middle 15th percentile. There was no physical activity by *APOE* interaction.

### Alzheimer's Disease Pathology

Eight of the studies investigated AD pathology, with all eight assessing Aβ and two also assessing tau (see Table 3 for AD pathology data).

**Table 3:** Associations between physical activity and Alzheimer's disease pathology.

**Outcome**	**Study**	**PA measurement**	**Outcome measure**	**PA association with outcome**	***APOE* association with outcome**	**PA x *APOE* interaction association with outcome**	**Association of PA with outcome according to** ***APOE*** **status**	
							**e4–**	**e4+**
Brain Aβ	Brown et al. (2013)	Total MET minutes/week T3: 7590 (*n* = 182) T2: 3206 (*n* = 182) T1: 1212 (*n* = 182)	PiB-PET Aβ (SUVR)	T3: 1.30 (0.31) T2: 1.44 (0.48) T1: 1.47 (0.38) *F* = 2.06, *p* = 0.09	*B* = 0.16, *p* = 0.03	NA. Analyses stratified by *APOE*	T3: 1.31 (0.35) T2: 1.32 (0.37) T1: 1.34 (0.30) *F* = 0.10, *p* = 0.90	T3: 1.28 (0.30) T2: 1.62 (0.57) T1: 1.65 (0.43) *F* = 3.68, *p* = 0.03
	de Souto Barreto et al. (2015)	Total MET-minutes/week	Florbetapir PET Aβ (SUVR)^a^	High SUVR: 1345 Low SUVR: 1194 *t* = NR *p* = 0.17 (Analysis reversed to assess PA level depending on high vs. low SUVR status)^a^	High SUVR: 38.1% e4+ Low SUVR: 14% e4+ *t* = NR, *p* < .001 (Analysis reversed to assess *APOE* status depending on high vs. low SUVR status)^a^	NA. Analyses stratified by *APOE*	rho = 0.09, *p* = 0.24	rho = 0.04, *p* = 0.77
	Head et al. (2012)	Total MET-hours/week (past 10 years): High exercise: ≥7.5 (*n* = 38) Low exercise: <7.5 (*n* = 125)	PiB-PET Aβ (MCBP)	High exercise: 0.01 (0.06) Low exercise: 0.09 (0.20) β = −0.188, *p* < 0.001	e4–: 0.03 (0.10) e4+: 0.16 (0.26) β = 0.371, *p* < 0.001	β = −0.236, *p* = 0.002	High exercise: 0.0097 (0.0600) Low exercise: 0.0301 (0.1065) *t* = NR, *p* > 0.05	High exercise: 0.0234 (0.0497) Low exercise: 0.2027 (0.2853) *t* = NR, *p* < 0.05
	Jeon et al. (2020)	Total MET-hours/week (midlife leisure activities)	PiB-PET Aβ (SUVR)	β = −0.001, *p* = 0.575	β = 0.174, *p* < 0.001	β = 0.001, *p* = 0.688	β = −0.079, *p* = 0.248	β = 0.017, *p* = 0.168
	Liang et al. (2010)	Total MET-hours/week (past 10 years): High exercise (≥7.5) (*n* = 11) Low exercise (<7.5) (*n* = 43)	PiB-PET Aβ (MCBP)	High exercise: 0.02 (0.03) Low exercise: 0.10 (0.16) *t* = 1.106, *p* = 0.274 *t* = 3.477, *p* = 0.001^b^	e4–: 0.048 e4+: 0.214 *t* = 2.070, *p* = 0.055	NA. Analyses stratified by *APOE*	High exercise: 0.0225 (0.0333) Low exercise: 0.0567 (0.0902) *t* = 1.6, *p* = 0.119	High exercise:−0.021 (NA)^c^ Low exercise: 0.2294 (0.3213) *t* = NA, *p* = NA
	Stojanovic et al. (2020)	Total MET-hours/week (past 10 years): High exercise: ≥7.5 (*n* = 59) Low exercise: <7.5 (*n* = 122)	PiB-PET Aβ (MCBP)	Group means = NR *t* = NR, *p* = NR	e4+ > e4– *t* = NR, *p* < .001	*B* = NR, *p* = NR	High exercise: 0.0596 (0.117) Low exercise: 0.0900 (0.174) *t* = NR, *p* = NR	High exercise: 0.1875 (0.208) Low exercise: 0.2158 (0.230) *t* = NR, *p* = NR
	Vemuri et al. (2016)	MET scores from midlife (50–65 years)	PiB-PET Aβ (SUVR)	*B* = NR, *p* > .05	*B* = −0.1398, *p* < 0.001	*B* = NR, *p* > 0.05	*r* = 0.01, *p* = 0.86^d^	*r* = −0.06, *p* = 0.54^d^
CSF Aβ	Head et al. (2012)	Total MET-hours/week (past 10 years): High exercise: ≥7.5 (*n* = 35) Low exercise: <7.5 (*n* = 130)	CSF Aβ_42_ (pg/mL)	High exercise: 710 (229) Low exercise: 620 (212) β = 0.181, *p* = 0.008	e4–: 692 (217) e4+: 536 (181) β = −0.346, *p* < 0.001	β = 0.024, *p* = 0.41	High exercise: 772.09 (239.52) Low exercise: 671.73 (207.414) *t* = NR, *p* = NR	High exercise: 604.85 (168.883) Low exercise: 514.97 (181.013) *t* = NR, *p* = NR
	Liang et al. (2010)	Total MET-hours/week (past 10 years): High exercise: ≥7.5 (*n* = 10) Low exercise: <7.5 (*n* = 46)	CSF fluid Aβ_42_ (pg/mL)	High exercise: 739 (217) Low exercise: 600 (185) *t* = 1.680, *p* = 0.099 *t* = 2.082, *p* = 0.042^b^	e4–: 679 e4+: 564 *t* = 2.024, *p* = 0.048	NA. Analyses stratified by *APOE*	High exercise: 758.66 (241.696) Low exercise: 655.1 (210.375) *t* = 0.944, *p* = 0.352 *t* = 1.319, *p* = 0.197^b^	High exercise: 660.17 (29.465) Low exercise: 554.15 (185.934) *t* = NR, *p* = NR
	Stojanovic et al. (2020)	Total MET-hours/week (past 10 years): High exercise: ≥7.5 (*n* = 33) Low exercise: <7.5 (*n* = 62)	CSF Aβ_42_ (pg/mL)	Group means = NR *t* = NR, *p* = NR	e4+ < e4– *t* = NR, *p* = 0.002	*B* = NR, *p* = NR	High exercise: 1260.588 (285.54) Low exercise: 1253.585 (349.45) *t* = NR, *p* = NR	High exercise: 927.138 (309.71) Low exercise: 1055.36 (396.09) *t* = NR, *p* = NR
Blood plasma Aβ	Brown et al. (2013)	Total MET minutes/week T3: 7700 (*n* = 38) T2: 3444 (*n* = 39) T1: 1359 (*n* = 39)	Plasma Aβ_42/40_ INNO-BIA fasting blood assay (pg/mL)	T3: 0.20 (0.06) T2: 0.21 (0.06) T1: 0.22 (0.07) *F* = 5.48, *p* = 0.003	*B* = 0.007, *p* = 0.27	NA. Analyses stratified by *APOE*	T3: Aβ_42/40_ = 0.19 (0.06) T2: Aβ_42/40_ = 0.21 (0.06) T1: Aβ_42/40_ = 0.22 (0.07) *F* = 6.77, *p* = 0.001	T3: 0.21 (0.06) T2: 0.21 (0.06) T1: 0.22 (0.06) *F* = 0.26, *p* = 0.77
			Plasma Aβ_42/40_ ELISA fasting blood assay (pg/mL)	T3: 0.39 (0.14) T2: 0.44 (0.17) T1: 0.41 (0.15) *F* = 2.87, *p* = 0.06	*B* = 0.003, *p* = 0.83	NA. Analyses stratified by *APOE*	T3: Aβ_42/40_ = 0.39 (0.16) T2: Aβ_42/40_ = 0.45 (0.18) T1: Aβ_42/40_ = 0.40 (0.13) *F* = 6.45, *p* = 0.002	T3: 0.38 (0.13) T2: 0.41 (0.13) T1: 0.46 (0.20) *F* = 2.55, *p* = 0.08
	Tsai et al. (2021)	${\text{VO}}_{2}^{\text{max}}$ (estimated from Rockport Fitness Walking Test)	Plasma Aβ_42_ (pg/mL)	*r* = NR, *p* < 0.05	e4–: 28.82 (35.34) e4+: 30.96 (38.63) *p* = 0.848	NA. Analyses stratified by *APOE*	*r* = −0.37, *p* = 0.086	*r* = −0.45, *p* = 0.035
Red blood cell Aβ	Piccarducci et al. (2019)	Minutes PA/week Active: ≥150 (*n* = 21) Non-active: <150 (*n* = 21)	Aβ in erythrocytes ELISA assay (ng/mg)	Group means = NR *t* = NR, *p* = NR	e4–: 12.4 (8.82) e4+: 18.0 (8.65) *t* = NR, *p* = 0.021	NA. Analyses stratified by *APOE*	Active: Aβ = 5.70 (2.77) Non-active: = 19.6 (7.19) *t* = NR, *p* < .001	Active: 12.1 (4.37) Non-active: 22.7 (8.24) *t* = NR, *p* = 0.009
CSF tau	Liang et al. (2010)	Total MET-hours/week (past 10 years): High exercise: ≥7.5 (*n* = 10) Low exercise: <7.5 (*n* = 46)	CSF tau (pg/mL)	High exercise: 263 (58) Low exercise: 282 (152) *t* = 0.140, *p* = 0.890	e4–: 264 e4+ 303 *t* = 1.021, *p* = 0.312	NA. Analyses stratified by *APOE*	High exercise: 252.87 (56.69) Low exercise: 266.85 (147.21) *t* = NR, *p* = NR	High exercise: 302 (57.98) Low exercise: 302.98 (159.01) *t* = NR, *p* = NR
	Stojanovic et al. (2020)	Total MET-hours/week (past 10 years): High exercise: ≥7.5 (*n* = 33) Low exercise: <7.5 (*n* = 62)	CSF tau (pg/mL)	Group means = NR *t* = NR, *p* = NR	e4+ > e4– *t* = NR, *p* = 0.004	*B* = NR, *p* = NR	High exercise: 243.820 (120.11) Low exercise: 265.09 (167.08) *t* = NR, *p* > 0.05^e^	High exercise: 321.175 (130.76) Low exercise: 367.803 (171.79) *t* = NR, *p* > 0.05^e^
CSF phosphorylated tau	Liang et al. (2010)	Total MET-hours/week (past 10 years): High exercise: ≥7.5 (*n* = 10) Low exercise: <7.5 (*n* = 46)	CSF ptau_181_ (pg/mL)	High exercise: 49 (13) Low exercise: 54 (25) *t* = 0.332, *p* = 0.743	e4–: 50 e4+: 58 *t* = 1.337, *p* = 0.187	NA. Analyses stratified by *APOE*	High exercise: 46.75 (12.56) Low exercise: 50.83 (23.87) *t* = NR, *p* = NR	High exercise: 57.46 (10.54) Low exercise: 58.54 (26.62) *t* = NR, *p* = NR
	Stojanovic et al. (2020)	Total MET-hours/week (past 10 years): High exercise: ≥7.5 (*n* = 33) Low exercise: <7.5 (*n* = 62)	CSF ptau_181_ (pg/mL)	Group means = NR *t* = NR, *p* = NR	e4+ > e4– *t* = NR, *p* = 0.033	*B* = NR, *p* = NR	High exercise: 45.265 (18.46) Low exercise: 50.381 (28.96) *t* = NR, *p* > 0.05^e^	High exercise: 58.983 (23.30) Low exercise: 60.095 (22.56) *t* = NR, *p* > 0.05^e^

*Aβ, Amyloid Beta; APOE, Apolipoprotein E; CSF, Cerebrospinal fluid; e4–, No APOE e4 alleles; e4+, Carrier of one or two APOE e4 alleles (includes e2e4 genotype unless stated otherwise); MCBP, Mean cortical binding potential; MET, Metabolic equivalent of task; NA, Not applicable; NR, Not reported; PET, Positron emission tomography; PA, Physical activity; PiB, Pittsburgh compound B; SUVR, Standardised uptake value ratio. Additional data not included in the original publications are included in this review for Liang et al. (2010), Head et al. (2012), Brown et al. (2013), Vemuri et al. (2016), Jeon et al. (2020), Stojanovic et al. (2020), and Tsai et al. (2021)*.

a *Association between PA/APOE and SUVR was assessed in subgroups according to SUVR with a 1.10 threshold. High SUVR > 1.10, low SUVR ≤ 1.10*.

b *With outlier removed*.

c *Only one participant in group*.

d *Study reversed Aβ measure so that higher values represented lower Aβ burden. The Pearson's r shown here is reversed so that a positive correlation represents an association where Aβ increases as physical activity increases*.

e *Significance test calculated from conversion to Pearson's r using Campbell Collaboration calculator*.

#### Amyloid Beta

**Figure 4:**
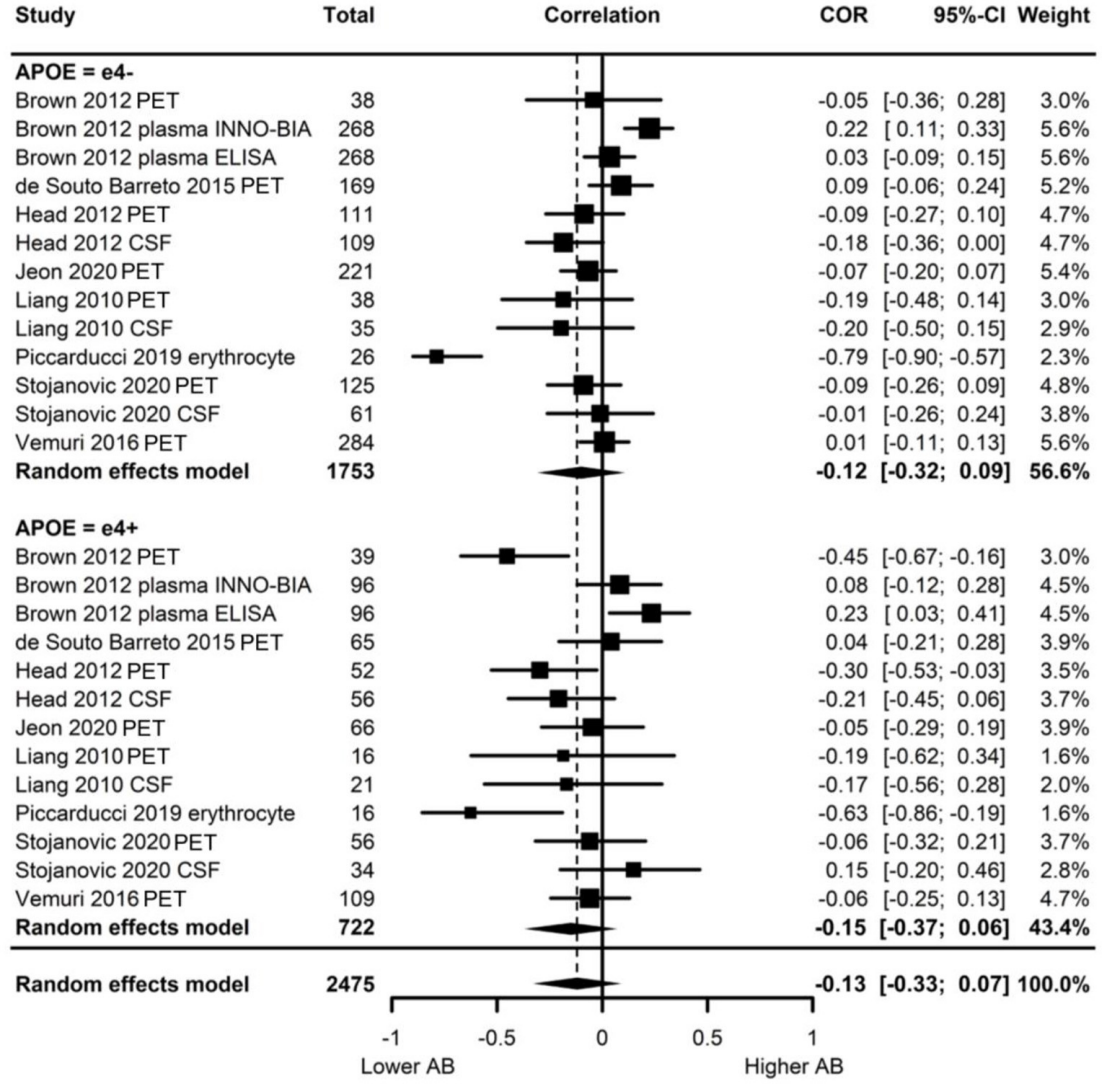
Forest plot indicating the association between physical activity and Aβ with carrier (e4+) and non-carrier (e4–) subgroups. Subgroup moderation test indicated no significant difference between *APOE* groups (*p* = 0.54).

Of the eight studies assessing Aβ, two provided evidence of moderation of the physical activity-Aβ association by *APOE*. All effect sizes were available, resulting in a full meta-analysis on the eight studies. AICs and BICs indicated that the multilevel model was a significantly better fit than the standard model (*p* = 0.01, see Supplementary Table 2 for model fit statistics). Physical activity was not significantly associated with Aβ (*r* = −0.13, *p* = 0.19), and this was also the case in e4 carriers (*r* = −0.15, *p* = 0.15) and non-carriers (*r* = −0.12, *p* = 0.24) separately (Figure 4). The moderation test indicated that there was no significant difference between *APOE* subgroups [*F*_(1, 24)_ = 0.38, *p* = 0.54].

Visual inspection of the funnel plot (Supplementary Figure 1) indicated possible publication bias with smaller studies more likely to be published if demonstrating an association between physical activity and reduced Aβ, however, this bias did not differ by *APOE* status. Heterogeneity was high (I^2^ = 86.5%), with 80.0% of the heterogeneity at the between cluster level. No sensitivity analyses to explain the heterogeneity were identified.

#### Tau

Of the two studies assessing tau, one (Liang et al., 2010) found no main effects of physical activity and *APOE* on either tau or phosphorylated tau, and it did not investigate outcomes stratified by *APOE*. The other (Stojanovic et al., 2020) found a main effect of *APOE*, with e4 carriers having higher levels of both tau and phosphorylated tau. However, physical activity was not associated with tau or phosphorylated tau in e4 carriers or non-carriers.

### Brain Structure

Of the six studies which assessed brain structure, five assessed GM volume, three assessed WM volume, one assessed WM integrity and two assessed cerebrovascular health (see Table 4 for brain structure data).

**Table 4:** Associations between physical activity and brain structure.

**Outcome**	**Study**	**PA measurement**	**Outcome measure**	**PA association with outcome**	***APOE* association with outcome**	**PA x *APOE* interaction association with outcome**	**Association of PA with outcome according to** ***APOE*** **status**	
							**e4–**	**e4+**
GM volume	Boots et al. (2015)	Graded Exercise Testing validated Measure of Cardiorespiratory Fitness	Hippocampus	*B* = 37.87, SE = 14.65, *p* = 0.010	*B* = NR, *p* = NR	*B* = NR, *p* ≥ 0.139	*B* = NR, *p* = NR	*B* = NR, *p* = NR
			Amygdala	*B* = 16.52, SE = 7.41, *p* = 0.026	*B* = NR, *p* = NR	*B* = NR, *p* ≥ 0.139	*B* = NR, *p* = NR	*B* = NR, *p* = NR
			Posterior cingulate	*B* = −4.59, SE = 13.64, *p* = 0.737	*B* = NR, *p* = NR	*B* = NR, *p* ≥ 0.139	*B* = NR, *p* = NR	*B* = NR, *p* = NR
			Cingulate isthmus	*B* = 11.58, SE = 11.25, *p* = 0.304	*B* = NR, *p* = NR	*B* = NR, *p* ≥ 0.139	*B* = NR, *p* = NR	*B* = NR, *p* = NR
			Parahippocampus	*B* = 13.21, SE = 8.86, *p* = 0.137	*B* = NR, *p* = NR	*B* = NR, *p* ≥ 0.139	*B* = NR, *p* = NR	*B* = NR, *p* = NR
			Entorhinal	*B* = 16.63, SE = 9.46, *p* = 0.080	*B* = NR, *p* = NR	*B* = NR, *p* ≥ 0.139	*B* = NR, *p* = NR	*B* = NR, *p* = NR
			Fusiform	*B* = 164.41, SE = 32.28, *p* < 0.001	*B* = NR, *p* = NR	*B* = NR, *p* ≥ 0.139	*B* = NR, *p* = NR	*B* = NR, *p* = NR
			Caudal anterior cingulate	*B* = 4.07, SE = 12.73, *p* = 0.749	*B* = NR, *p* = NR	*B* = NR, *p* ≥ 0.139	*B* = NR, *p* = NR	*B* = NR, *p* = NR
			Rostral anterior cingulate	*B* = 5.73, SE = 12.17, *p* = 0.638	*B* = NR, *p* = NR	*B* = NR, *p* ≥ 0.139	*B* = NR, *p* = NR	*B* = NR, *p* = NR
			Caudal middle frontal	*B* = 21.31, SE = 33.28, *p* = 0.522	*B* = NR, *p* = NR	*B* = NR, *p* ≥ 0.139	*B* = NR, *p* = NR	*B* = NR, *p* = NR
			Rostral middle frontal	*B* = 113.31, SE = 51.35, *p* = 0.028	*B* = NR, *p* = NR	*B* = NR, *p* ≥ 0.139	*B* = NR, *p* = NR	*B* = NR, *p* = NR
			Inferior parietal	*B* = 108.92, SE = 51.04, *p* = 0.034	*B* = NR, *p* = NR	*B* = NR, *p* ≥ 0.139	*B* = NR, *p* = NR	*B* = NR, *p* = NR
			Precuneus	*B* = 71.89, SE = 33.51, *p* = 0.033	*B* = NR, *p* = NR	*B* = NR, *p* ≥ 0.139	*B* = NR, *p* = NR	*B* = NR, *p* = NR
			Supramarginal	*B* = 162.17, SE = 41.21, *p* < 0.001	*B* = NR, *p* = NR	*B* = NR, *p* ≥ 0.139	*B* = NR, *p* = NR	*B* = NR, *p* = NR
	Gu et al. (2020)	MET-minutes/week: High active: ≥1025 (*n* = 357) Moderately active: 450–1,024 (*n* = 382) Low active: 1–449 (*n* = 346) Inactive: 0 (*n* = 304)	Total GM	*B* = NR, *p* < 0.001	*B* = NR, *p* = NR	NA. Analyses stratified by *APOE*	High active: 548.6 (52.96) Moderately active: 537.3 (53.12) Low active: 524.6 (53.37) Inactive: 517.5 (53.23) *t* = NR, *p* = NR	High active: 548.7 (49.16) Moderately active: 543.5 (49.84) Low active: 537.5 (50.06) Inactive: 521.8 (48.99) *t* = NR, *p* = NR
			Hippocampus	*B* = NR, *p* = 0.32	*B* = NR, *p* = NR	NA. Analyses stratified by *APOE*	High active: 7.1 (0.90) Moderately active: 7.0 (0.90) Low active: 6.9 (0.90) Inactive: 6.8 (0.91) *t* = NR, *p* = NR	High active: 7.1 (0.82) Moderately active: 7.1 (0.84) Low active: 7.0 (0.84) Inactive: 6.6 (0.82) *t* = NR, *p* = NR
	Honea et al. (2009)	${\text{VO}}_{2}^{\text{peak}}$ (mL/kg/min)	Right inferior frontal gyrus	*r* = NR, *p* = 0.812	*B* = NR, *p* = NR	*B* = NR, *p* > 0.05	*r* = NR, *p* = NR	*r* = NR, *p* = NR
	Smith et al. (2014)	High active: leisure time activity including moderate to vigorous activity of >15 min on >3 days per week (*n* = 46) Low active: no PA or low intensity activity such as slow walking or light chores ≤ 2 days per week (*n* = 51)	Hippocampal 18-month change	Group means = NR *F* = NR, *p* = 0.314	Group means = NR *F* = NR, *p* = 0.082	*F* = NR, *p* = 0.024	High active: −0.82% (3.60) Low active: 0.15% (3.49) *t* = NR, *p* = NR	High active: −0.41% (3.61) Low active: −2.91% (3.79) *t* = NR, *p* = NR
			Thalamus 18-month change	Group means = NR *F* = NR, *p* = 0.228	Group means = NR *F* = NR, *p* = 0.677	*F* = NR, *p* = 0.351	High active: −2.06% (2.72) Low active: −0.85% (3.05) *t* = NR, *p* = NR	High active: −1.77% (2.23) Low active: −1.61% (2.32) *t* = NR, *p* = NR
			Caudate 18-month change	Group means = NR *F* = NR, *p* = 0.171	Group means = NR *F* = NR, *p* = 0.177	*F* = NR, *p* = 0.759	High active: −0.48% (3.58) Low active: −1.24% (3.69) *t* = NR, *p* = NR	High active: −1.23% (2.73) Low active: −2.44% (3.42) *t* = NR, *p* = NR
			Amygdala 18-month change	Group means = NR *F* = NR, *p* = 0.812	Group means = NR *F* = NR, *p* = 0.465	*F* = NR, *p* = 0.777	High active: 0.59% (7.68) Low active: 1.40% (8.40) *t* = NR, *p* = NR	High active: −0.10% (6.86) Low active: −0.17% (4.77) *t* = NR, *p* = NR
			Caudal middle frontal gyrus 18-month change	Group means = NR *F* = NR, *p* = 0.614	Group means = NR *F* = NR, *p* = 0.593	*F* = NR, *p* = 0.715	High active: 0.92% (3.42) Low active: 1.04% (3.95) *t* = NR, *p* = NR	High active: 0.11% (4.74) Low active: 0.89% (5.08) *t* = NR, *p* = NR
			Pre-central gyrus 18- month change	Group means = NR *F* = NR, *p* = 0.711	Group means = NR *F* = NR, *p* = 0.375	*F* = NR, *p* = 0.924	High active: −0.09% (3.45) Low active: −0.51% (4.31) *t* = NR, *p* = NR	High active: −0.97% (4.68) Low active: −1.21% (4.48) *t* = NR, *p* = NR
			Total GM 18-month change	Group means = NR *F* = NR, *p* = 0.340	Group means = NR *F* = NR, *p* = 0.551	*F* = NR, *p* = 0.421	High active: −0.39% (2.27) Low active: −0.49% (3.13) *t* = NR, *p* = NR	High active: −0.26% (3.27) Low active: −1.37% (3.18) *t* = NR, *p* = NR
	Vemuri et al. (2016)	MET scores from midlife (50–65 years)	Hippocampus	*B* = NR, *p* > 0.05	*B* = NR, *p* > 0.05	*B* = NR, *p* > 0.05	*r* = −0.01, *p* = 0.92	*r* = 0.15, *p* = 0.13
WM volume	Gu et al. (2020)	MET-minutes/week: High active: ≥1,025 (*n* = 357) Moderately active: 450–1,024 (*n* = 382) Low active: 1–449 (*n* = 346) Inactive: 0 (*n* = 304)	Total WM	*B* = NR, *p* = 0.02	Group means = NR, *t* = NR, *p* = NR	NA. Analyses stratified by *APOE*	High active: 407.8 (53.21) Moderately active: 391.2 (53.39) Low active: 384.4 (53.62) Inactive: 376.2 (53.49) *r* = 0.28 (0.19; 0.37)^a^	High active: 400.5 (52.52) Moderately active: 396.5 (53.25) Low active: 394.5 (53.49) Inactive: 383 (52.35) *r* = 0.16 (0.01; 0.30)^a^
	Honea et al. (2009)	${\text{VO}}_{2}^{\text{peak}}$ (mL/kg/min)	Right inferior occipital gyrus	*r* = NR, *p* = 0.771	Group means = NR *t* = NR, *p* = NR	β = NR, *p* > 0.05	*r* = NR, *p* = NR	*r* = NR, *p* = NR
			Left middle occipital gyrus	*r* = NR, *p* = 0.996	Group means = NR *t* = NR, *p* = NR	β = NR, *p* > 0.05	*r* = NR, *p* = NR	*r* = NR, *p* = NR
			Left lentiform nucleus gyrus	*r* = NR, *p* = 0.996	Group means = NR *t* = NR, *p* = NR	β = NR, *p* > 0.05	*r* = NR, *p* = NR	*r* = NR, *p* = NR
			Left lingual gyrus	*r* = NR, *p* = 1	Group means = NR *t* = NR, *p* = NR	β = NR, *p* > 0.05	*r* = NR, *p* = NR	*r* = NR, *p* = NR
	Smith et al. (2014)	High active: leisure time activity including moderate to vigorous activity of >15 min on >3 days per week (*n* = 46) Low active: no PA or low intensity activity such as slow walking or light chores ≤ 2 days per week (*n* = 51)	Total cortical WM volume 18-month change	Group means = NR *F* = NR, *p* = 0.178	Group means = NR *F* = NR, *p* = 0.486	*F* = NR, *p* = 0.252	High active: −1.43% (2.66) Low active: −0.37% (2.07) *r* = −0.21 (−0.44; 0.04)^b^	High active: −0.65% (1.42) Low active: −0.56% (1.37) *r* = −0.03 (−0.33; 0.27)^b^
WM integrity	Smith et al. (2016)	High active: leisure time activity including moderate to vigorous activity of >15 min on >3 days per week (*n* = 41) Low active: no PA or low intensity activity such as slow walking or light chores ≤ 2 days per week (*n* = 47)	FA left superior longitudinal fasciculus	Group means = NR *F* = NR, *p* = 0.493	Group means = NR *F* = NR, *p* = 0.174	*F* = NR, *p* = 0.0016^c^	High active: 0.442 (0.029) Low active: 0.432 (0.027) *t* = NR, *p* = 0.044	High active: 0.437 (0.021) Low active: 0.457 (0.021) *t* = NR, *p* = 0.012
			FA right superior longitudinal fasciculus	Group means = NR *F* = NR, *p* = 0.854	Group means = NR *F* = NR, *p* = 0.359	*F* = NR, *p* = 0.0443^d^	High active: 0.434 (0.029) Low active: 0.427 (0.028) *t* = NR, *p* > 0.1	High active: 0.433 (0.015) Low active: 0.441 (0.020) *t* = NR, *p* > 0.1
			FA left sagittal stratum	Group means = NR *F* = NR, *p* = 0.158	Group means = NR *F* = NR, *p* = 0.602	*F* = NR, *p* = 0.0001^c^	High active: 0.483 (0.026) Low active: 0.472 (0.031) *t* = NR, *p* = 0.017	High active: 0.468 (0.032) Low active: 0.501 (0.028) *t* = NR, *p* = 0.0003
			FA right sagittal stratum	Group means = NR *F* = NR, *p* = 0.378	Group means = NR *F* = NR, *p* = 0.103	*F* = NR, *p* = 0.0005^c^	High active: 0.494 (0.024) Low active: 0.481 (0.036) *t* = NR, *p* = 0.031	High active: 0.487 (0.028) Low active: 0.515 (0.030) *t* = NR, *p* = 0.004
			FA left uncinate fasciculus	Group means = NR *F* = NR, *p* = 0.913	Group means = NR *F* = NR, *p* = 0.219	*F* = NR, *p* = 0.069	High active: 0.433 (0.055) Low active: 0.416 (0.054) *t* = NR, *p* > 0.1	High active: 0.433 (0.047) Low active: 0.451 (0.057) *t* = NR, *p* > 0.1
			FA right uncinate fasciculus	Group means = NR *F* = NR, *p* = 0.335	Group means = NR *F* = NR, *p* = 0.709	*F* = NR, *p* = 0.034^d^	High active: 0.458 (0.055) Low active: 0.429 (0.059) *t* = NR, *p* = 0.015	High active: 0.446 (0.043) Low active: 0.457 (0.041) *t* = NR, *p* > 0.1
			FA left cingulate gyrus	Group means = NR *F* = NR, *p* = 0.980	Group means = NR *F* = NR, *p* = 0.954	*F* = NR, *p* = 0.0033^c^	High active: 0.444 (0.032) Low active: 0.425 (0.038) *t* = NR, *p* = 0.019	High active: 0.426 (0.030) Low active: 0.448 (0.036) *t* = NR, *p* = 0.051
			FA right cingulate gyrus	Group means = NR *F* = NR, *p* = 0.405	Group means = NR *F* = NR, *p* = 0.851	*F* = NR, *p* = 0.0092^c^	High active: 0.415 (0.036) Low active: 0.405 (0.034) *t* = NR, *p* > 0.1	High active: 0.398 (0.030) Low active: 0.423 (0.038) *t* = NR, *p* = 0.026
			FA left cingulum (hippocampal projection)	Group means = NR *F* = NR, *p* = 0.809	Group means = NR *F* = NR, *p* = 0.698	*F* = NR, *p* = 0.038^d^	High active: 0.320 (0.039) Low active: 0.314 (0.038) *t* = NR, *p* > 0.1	High active: 0.317 (0.040) Low active: 0.332 (0.038) *t* = NR, *p* > 0.1
			FA right cingulum (hippocampal projection)	Group means = NR *F* = NR, *p* = 0.459	Group means = NR *F* = NR, *p* = 0.751	*F* = NR, *p* = 0.044^d^	High active: 0.331 (0.030) Low active: 0.318 (0.039) *t* = NR, *p* = 0.03	High active: 0.327 (0.045) Low active: 0.335 (0.033) *t* = NR, *p* > 0.1
			FA left fornix	Group means = NR *F* = NR, *p* = 0.002	Group means = NR *F* = NR, *p* = 0.531	*F* = NR, *p* = 0.021^c^	High active: 0.422 (0.035) Low active: 0.433 (0.026) *t* = NR, *p* > 0.1	High active: 0.417 (0.035) Low active: 0.453 (0.037) *t* = NR, *p* = 0.001
			FA right fornix	Group means = NR *F* = NR, *p* = 0.483	Group means = NR *F* = NR, *p* = 0.558	*F* = NR, *p* = 0.0042^c^	High active: 0.440 (0.030) Low active: 0.429 (0.031) *t* = NR, *p* = 0.08	High active: 0.420 (0.040) Low active: 0.445 (0.043) *t* = NR, *p* = 0.02
			FA body of corpus callosum	Group means = NR *F* = NR, *p* = 0.768	Group means = NR *F* = NR, *p* = 0.165	*F* = NR, *p* = 0.072	High active: 0.531 (0.052) Low active: 0.518 (0.045) *t* = NR, *p* > 0.1	High active: 0.508 (0.050) Low active: 0.521 (0.035) *t* = NR, *p* > 0.1
			FA genu of corpus callosum	Group means = NR *F* = NR, *p* = 0.413	Group means = NR *F* = NR, *p* = 0.685	*F* = NR, *p* = 0.045^d^	High active: 0.586 (0.046) Low active: 0.571 (0.035) *t* = NR, *p* = 0.026	High active: 0.575 (0.032) Low active: 0.582 (0.031) *t* = NR, *p* > 0.1
			FA splenium of corpus callosum	Group means = NR *F* = NR, *p* = 0.239	Group means = NR *F* = NR, *p* = 0.823	*F* = NR, *p* = 0.188	High active: 0.722 (0.023) Low active: 0.711 (0.028) *t* = NR, *p* = 0.05	High active: 0.719 (0.019) Low active: 0.719 (0.028) *t* = NR, *p* > 0.1
			MD left superior longitudinal fasciculus	Group means = NR *F* = NR, *p* = 0.989	Group means = NR *F* = NR, *p* = 0.031	*F* = NR, *p* = 0.0013^c^	High active: 0.763 (0.033) Low active: 0.780 (0.036) *t* = NR, *p* = 0.01	High active: 0.763 (0.034) Low active: 0.742 (0.026) *t* = NR, *p* = 0.034
			MD right superior longitudinal fasciculus	Group means = NR *F* = NR, *p* = 0.935	Group means = NR *F* = NR, *p* = 0.241	*F* = NR, *p* = 0.0020^c^	High active: 0.764 (0.034) Low active: 0.779 (0.035) *t* = NR, *p* = 0.012	High active: 0.770 (0.035) Low active: 0.750 (0.027) *t* = NR, *p* = 0.046
			MD left sagittal stratum	Group means = NR *F* = NR, *p* = 0.34	Group means = NR *F* = NR, *p* = 0.973	*F* = NR, *p* = 0.0027^c^	High active: 0.850 (0.037) Low active: 0.863 (0.039) *t* = NR, *p* = 0.091	High active: 0.871 (0.052) Low active: 0.837 (0.034) *t* = NR, *p* = 0.01
			MD right sagittal stratum	Group means = NR *F* = NR, *p* = 0.418	Group means = NR *F* = NR, *p* = 0.884	*F* = NR, *p* = 0.0036^c^	High active: 0.835 (0.040) Low active: 0.849 (0.039) *t* = NR, *p* = 0.086	High active: 0.857 (0.047) Low active: 0.825 (0.035) *t* = NR, *p* = 0.015
			MD left uncinate fasciculus	Group means = NR *F* = NR, *p* = 0.426	Group means = NR *F* = NR, *p* = 0.362	*F* = NR, *p* = 0.070	High active: 0.827 (0.046) Low active: 0.848 (0.052) *t* = NR, *p* = 0.041	High active: 0.849 (0.037) Low active: 0.840 (0.043) *t* = NR, *p* > 0.1
			MD right uncinate fasciculus	Group means = NR *F* = NR, *p* = 0.218	Group means = NR *F* = NR, *p* = 0.951	*F* = NR, *p* = 0.168	High active: 0.830 (0.042) Low active: 0.844 (0.051) *t* = NR, *p* = 0.043	High active: 0.833 (0.028) Low active: 0.835 (0.030) *t* = NR, *p* > 0.1
			MD left cingulate gyrus	Group means = NR *F* = NR, *p* = 0.959	Group means = NR *F* = NR, *p* = 0.303	*F* = NR, *p* = 0.0130^c^	High active: 0.762 (0.030) Low active: 0.774 (0.031) *t* = NR, *p* = 0.051	High active: 0.767 (0.034) Low active: 0.751 (0.027) *t* = NR, *p* = 0.096
			MD right cingulate gyrus	Group means = NR *F* = NR, *p* = 0.918	Group means = NR *F* = NR, *p* = 0.073	*F* = NR, *p* = 0.0096^c^	High active: 0.761 (0.035) Low active: 0.775 (0.033) *t* = NR, *p* = 0.031	High active: 0.761 (0.025) Low active: 0.745 (0.028) *t* = NR, *p* > 0.1
			MD left cingulum (hippocampal projection)	Group means = NR *F* = NR, *p* = 0.917	Group means = NR *F* = NR, *p* = 0.048	*F* = NR, *p* = 0.340	High active: 0.830 (0.055) Low active: 0.833 (0.052) *t* = NR, *p* > 0.1	High active: 0.857 (0.047) Low active: 0.847 (0.068) *t* = NR, *p* > 0.1
			MD right cingulum (hippocampal projection)	Group means = NR *F* = NR, *p* = 0.064	Group means = NR *F* = NR, *p* = 0.189	*F* = NR, *p* = 0.095	High active: 0.831 (0.042) Low active: 0.823 (0.038) *t* = NR, *p* > 0.1	High active: 0.849 (0.044) Low active: 0.822 (0.026) *t* = NR, *p* = 0.023
			MD left fornix	Group means = NR *F* = NR, *p* = 0.043	Group means = NR *F* = NR, *p* = 0.712	*F* = NR, *p* = 0.132	High active: 0.967 (0.094) Low active: 0.942 (0.053) *t* = NR, *p* > 0.1	High active: 0.964 (0.086) Low active: 0.915 (0.070) *t* = NR, *p* = 0.023
			MD right fornix	Group means = NR *F* = NR, *p* = 0.433	Group means = NR *F* = NR, *p* = 0.331	*F* = NR, *p* = 0.041^d^	High active: 0.985 (0.140) Low active: 0.989 (0.099) *t* = NR, *p* > 0.1	High active: 1.027 (0.133) Low active: 0.967 (0.117) *t* = NR, *p* = 0.067
			MD body of corpus callosum	Group means = NR *F* = NR, *p* = 0.199	Group means = NR *F* = NR, *p* = 0.83	*F* = NR, *p* = 0.105	High active: 0.961 (0.069) Low active: 0.984 (0.057) *t* = NR, *p* = 0.023	High active: 0.967 (0.071) Low active: 0.968 (0.071) *t* = NR, *p* > 0.1
			MD genu of corpus callosum	Group means = NR *F* = NR, *p* = 0.606	Group means = NR *F* = NR, *p* = 0.355	*F* = NR, *p* = 0.404	High active: 0.963 (0.063) Low active: 0.967 (0.059) *t* = NR, *p* > 0.1	High active: 0.969 (0.064) Low active: 0.970 (0.040) *t* = NR, *p* > 0.1
			MD splenium of corpus callosum	Group means = NR *F* = NR, *p* = 0.311	Group means = NR *F* = NR, *p* = 0.465	*F* = NR, *p* = 0.040^d^	High active: 0.804 (0.033) Low active: 0.826 (0.041) *t* = NR, *p* = 0.016	High active: 0.809 (0.046) Low active: 0.802 (0.037) *t* = NR, *p* > 0.1
WMH	Boots et al. (2015)	Graded Exercise Testing validated Measure of Cardiorespiratory Fitness	High v low total WMH volume	*B* = −0.33, OR = 0.72, *p* < 0.001	*B* = NR; OR = NR	*B* = NR, *p* ≥ 0.139	*B* = NR; OR = NR	*B* = NR; OR = NR
	Gu et al. (2020)	MET-minutes/week: High active: ≥1,025 (*n* = 357) Moderately active: 450–1,024 (*n* = 382) Low active: 1–449 (*n* = 346) Inactive: 0 (*n* = 304)	Total WMH volume	*B* = NR, *p* = 0.67	Group means = NR *t* = NR, *p* = NR	NA. Analyses stratified by *APOE*	High active: 4.47 (6.20) Moderately active: 4.23 (6.23) Low active: 4.56 (6.28) Inactive: 4.04 (6.21) *r* = 0.03 (−0.05; 0.12)^a^	High active: 4.09 (6.08) Moderately active: 4.96 (6.09) Low active: 5.33 (6.21) Inactive: 3.86 (6.04) *r* = 0.02 (−0.13; 0.16)^a^

*APOE, Apolipoprotein E; e4–, No APOE e4 alleles; e4+, Carrier of one or two APOE e4 alleles (includes e2e4 genotype unless stated otherwise); FA, Fractional anisotropy; GM, Grey matter; MD, Mean diffusivity; MET, Metabolic equivalent of task; NA, Not applicable; NR, Not reported; PA, Physical activity; OR, Odds ratio; WM, White matter; WMH, White matter hyperintensities. Additional data not included in the original publications are included in this review for Honea et al. (2009), Vemuri et al. (2016), and Gu et al. (2020)*.

a *Calculated using high active vs. inactive to Pearson's r using Campbell collaboration calculator*.

b *Calculated using high active vs. low active to Pearson's r using Campbell collaboration calculator*.

c *Significant following false discovery rate adjustment*.

d *Not significant following false discovery rate adjustment*.

#### Grey Matter Volume

**Figure 5:**
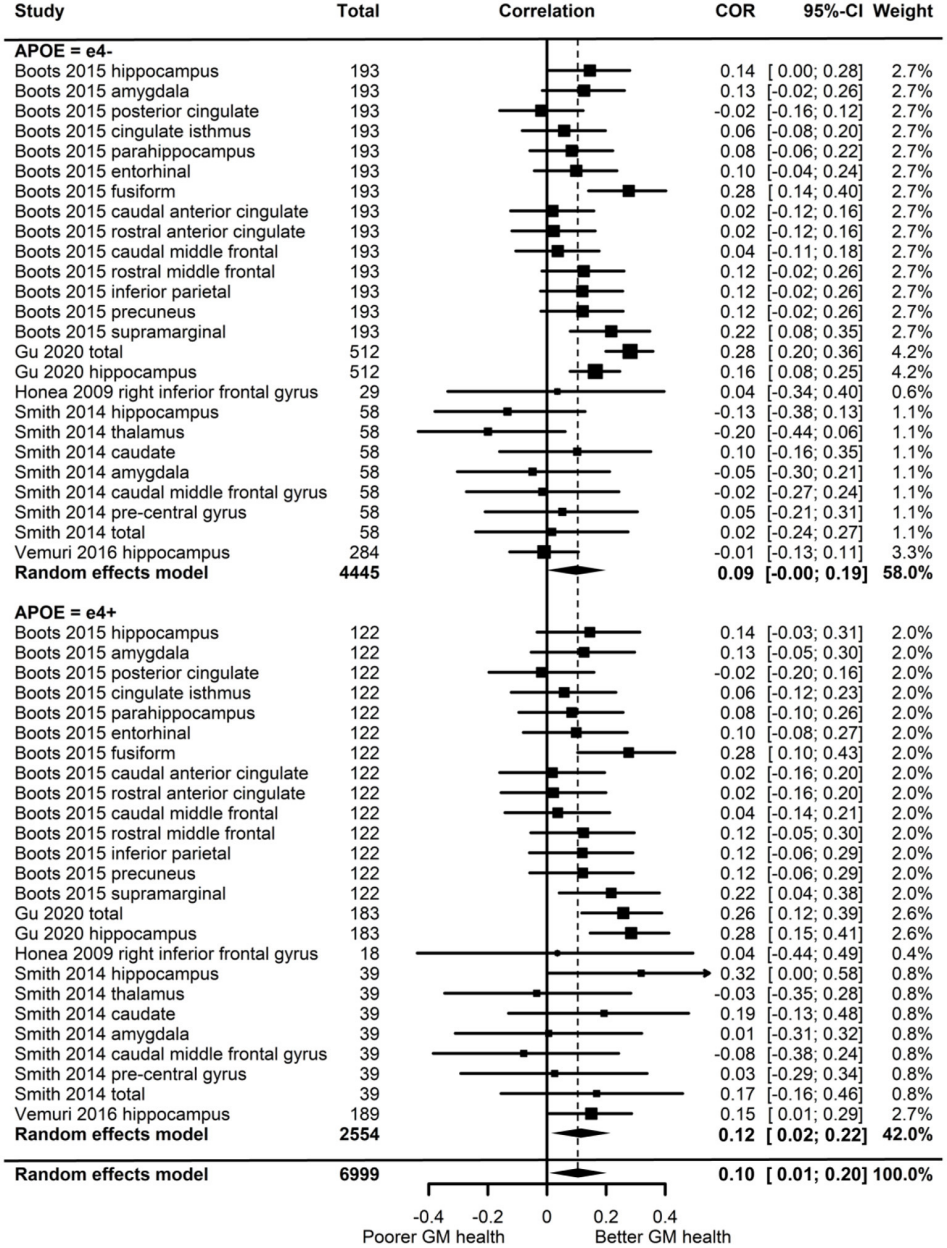
Forest plot indicating the association between physical activity and GM with carrier (e4+) and non-carrier (e4–) subgroups. Subgroup moderation test indicated no significant difference between *APOE* groups (*p* = 0.26).

Of the five studies which assessed grey matter volume, one provided evidence of *APOE* moderation of the physical activity-GM association. A meta-analysis was carried out with 25 effect sizes each for e4 carriers and non-carriers, 15 of which were substituted with the physical activity main effect from e4 carriers and non-carriers combined. AICs and BICs indicated that the full multilevel model was a significantly better fit than the standard model (*p* = 0.002; see Supplementary Table 2 for full model fit statistics). Physical activity was significantly associated with GM (*r* = 0.10, *p* = 0.03). A subgroup analysis revealed that physical activity was significantly associated with GM volume in e4 carriers (*r* = 0.12, *p* = 0.02) but not in e4 non-carriers (*r* = 0.09, *p* = 0.06) (Figure 5). However, the moderation test did not indicate a significant difference between e4 carriers and non-carriers [*F*_(1, 48)_ = 1.30, *p* = 0.26].

Visual inspection of the funnel plot (Supplementary Figure 1) did not suggest publication bias. Heterogeneity was moderate (I^2^ = 54.7%), with 49.1% of the heterogeneity at the between cluster level. *Post-hoc* investigation identified physical activity measurement and study design as possible sources of between cluster heterogeneity. One study (Smith et al., 2014) assessed the association between physical activity and eighteen-month change in GM volume, while the others assessed cross-sectional associations. A sensitivity analysis with the longitudinal study removed made minimal difference, with moderate overall heterogeneity (I^2^ = 58.3%) which was mostly at the between cluster level (I^2^ = 50.3%).

#### White Matter Volume

From the three studies which assessed WM volume, four of the six effect sizes for e4 carriers and non-carriers were not reported, and neither were their physical activity main effects. One study (Gu et al., 2020) only reported stratified *APOE* data and showed significant positive associations between physical activity and WM volume for both e4 carriers and non-carriers. Highly active e4 carriers had 17.5 cm^3^ higher WM volume compared to inactive e4 carriers, whereas active non-carriers had 31.6 cm^3^ higher WM volume compared to inactive non-carriers. Without an interaction test, it was not possible to confirm whether this difference was significant. The other two studies did investigate physical activity by *APOE* interactions. Honea et al. (2009) investigated WM in four regions, and Smith et al. (2014) investigated cortical WM change over 18 months. Both studies reported no difference in the association between physical activity and WM volume by *APOE* status.

#### White Matter Integrity

One study (Smith et al., 2016) assessed the association between physical activity and WM integrity. Of the 15 association and commissural fibre tracts assessed, there were seven significant interactions between physical activity and *APOE* on FA, and six significant interactions on MD. For e4 carriers, active participants unexpectedly demonstrated lower FA and higher MD. For non-carriers, active participants demonstrated the expected pattern of higher FA and lower MD. *Post-hoc* analysis by the author suggested that WM integrity measures were complicated due to crossing neural fibres, and the findings potentially indicated that e4 carriers benefit from physical activity as well as non-carriers (for more detail, see Smith et al., 2016).

#### Cerebrovascular Health

Two studies assessed cerebrovascular health indicated by WMH. One (Boots et al., 2015) demonstrated a main effect of physical activity on WMH, with more active participants having lower WMH (better cerebrovascular health). There was no significant physical activity by *APOE* interaction, suggesting that both e4 carriers and non-carriers benefited from being physically active. The other study (Gu et al., 2020) assessed the association between physical activity and WMH stratified by *APOE*, but neither e4 carriers nor non-carriers demonstrated an association between physical activity and cerebrovascular health.

### Brain Activation

Of the nine studies which assessed brain activation, four assessed activation during cognitive tasks, three assessed resting-state activation, and two assessed resting-state functional connectivity. These studies consisted of a mixture of fMRI, EEG and MEG, and studies which assessed activation were considered separately from studies which assessed connectivity (see Table 5 for brain activation data).

**Table 5:** Associations between physical activity and functional brain activation.

**Outcome**	**Study**	**PA measurement**	**Outcome measure**	**PA association with outcome**	***APOE* association with outcome**	**PA x *APOE* interaction association with outcome**	**Association of PA with outcome according to** ***APOE*** **status**	
							**e4–**	**e4+**
Task-related activation	Deeny et al. (2008)	High active: aerobic exercise ≥ 3 time per week (*n* = 14) Low active: no aerobic exercise (*n* = 9)	Right temporal region MEG activation (root mean square overall amplitude 0–600 ms)	Group differences = NR *F* = NR, *p* = NR	Group differences = NR *F* = NR, *p* = NR	*F* = 11.73, *p* = 0.003	High active: 31 (2) Low active: 36.5 (3) *t* = NR, *p* > 0.05	High active: 38 (3) Low active: 25 (4) *t* = NR, *p* < 0.05
			M170 MEG amplitude (root mean square peak between 130 and 250 ms)	High active > low active *F* = 5.48, *p* = 0.03	Group differences = NR *F* = NR, *p* > 0.05	*F* = NR, *p* > 0.05	Group differences = NR *t* = NR, *p* = NR	Group differences = NR *t* = NR, *p* = NR
			M170 MEG latency (ms)	Group differences = NR *F* = 3.5, *p* = 0.077	e4+ > than e4– *F* = 11.97, *p* = 0.003	*F* = 2.89, *p* = 0.105	High active: 171 (6) Low active: 173 (2) *t* = NR, *p* = NR	High active: 184 (12) Low active: 207 (12) *t* = NR, *p* = NR
	Smith et al. (2011)	High active: leisure time activity including moderate to vigorous activity of >15 min on >3 days per week (*n* = 34) Low PA: no PA or low intensity activity such as slow walking or light chores ≤ 2 days per week (*n* = 34)	BOLD response left BA 6, 8 BOLD response bilateral BA 6, 32	Group differences = NR *t* = NR, *p* = 0.145 High active > low active *t* = NR, *p* = 0.028	Group differences = NR *t* = NR, *p* = 0.178 Group differences = NR, *t* = NR, *p* = 0.779	*F* = NR, *p* = 0.022 *F* = NR, *p* = 0.067	High active: 0.15 (0.16) Low active: 0.21 (0.32) *t* = NR, *p* > 0.01 High active: −0.15 (0.19) Low active: −0.18 (0.38) *t* = NR, *p* = NR	High active: 0.40 (0.30) Low active: 0.14 (0.28) *t* = NR, *p* < 0.01 High active: −0.004 (0.29) Low active: −0.28 (0.21) *t* = NR, *p* = NR
			BOLD response left BA 6	High > low active *t* = NR, *p* = 0.045	Group differences NR *t* = NR, *p* = 0.096	*F* = NR, *p* = 0.286	High active: −0.20 (0.18) Low active: −0.26 (0.35) *t* = NR, *p* = NR	High active: −0.03 (0.21) Low active: −0.22 (0.25) *t* = NR, *p* = NR
			BOLD response left BA 8, 9	High active > low active *t* = NR, *p* = 0.044	Group differences = NR *t* = NR, *p* = 0.059	*F* = NR, *p* = 0.004	High active: −0.009 (0.37) Low active: 0.08 (0.45) *t* = NR, *p* > 0.01	High active: 0.46 (0.28) Low active: −0.02 (0.47) *t* = NR, *p* < 0.01
			BOLD response left BA 10, 32	Group differences = NR *t* = NR, *p* = 0.339	Group differences = NR *t* = NR, *p* = 0.331	*F* = NR, *p* < 0.001	High active: −0.02 (0.43) Low active: 0.84 (0.67) *t* = NR, *p* < 0.01	High active: 0.84 (1.14) Low active: 0.34 (0.66) *t* = NR, *p* > 0.01
			BOLD response right BA 44, 45	Group differences = NR *t* = NR, *p* = 0.136	Group differences = NR *t* = NR, *p* = 0.327	*F* = NR, *p* = 0.007	High active: −0.23 (0.31) Low active: −0.14 (0.40) *t* = NR, *p* > 0.01	High active: 0.04 (0.26) Low active: −0.27 (0.19) *t* = NR, *p* < 0.01
			BOLD response left BA 8, 9	Group differences = NR *t* = NR, *p* = 0.948	e4+ > e4– *t* = NR, *p* = 0.023	*F* = NR, *p* = 0.008	High active: −0.06 (0.73) Low active: 0.42 (0.75) *t* = NR, *p* > 0.01	High active: 0.85 (0.55) Low active: 0.34 (0.90) *t* = NR, *p* > 0.01
			BOLD response right BA 4, 6, 32	High active > low active *t* = NR, *p* = 0.039	e4+ > e4– *t* = NR, *p* = 0.016	*F* = NR, *p* = 0.180	High active: 0.003 (0.23) Low active: −0.04 (0.38) *t* = NR, *p* = NR	High active: 0.24 (0.16) Low active: 0.03 (0.23) *t* = NR, *p* = NR
			BOLD response left BA 7, 22, 39, 40	Group differences = NR *t* = NR, *p* = 0.052	Group differences = NR *t* = NR, *p* = 0.703	*F* = NR, *p* = 0.013	High active: 0.17 (0.18) Low active: 0.21 (0.37) *t* = NR, *p* > 0.01	High active: 0.37 (0.24) Low active: 0.07 (0.25) *t* = NR, *p* < 0.01
			BOLD response bilateral BA 7, 23, 29, 30	Group differences = NR *t* = NR, *p* = 0.332	Group differences = NR *t* = NR, *p* = 0.104	*F* = NR, *p* = 0.248	High active: 0.18 (0.27) Low active: 0.20 (0.52) *t* = NR, *p* = NR	High active: 0.45 (0.35) Low active: 0.24 (0.43) *t* = NR, *p* = NR
			BOLD response right BA 7	Group differences = NR *t* = NR, *p* = 0.845	Group differences = NR *t* = NR, *p* = 0.075	*F* = NR, *p* = 0.035	High active: −0.28 (0.13) Low active: −0.10 (0.37) *t* = NR, *p* > 0.01	High active: 0.03 (0.24) Low active: −0.12 (0.23) *t* = NR, *p* > 0.01
			BOLD response right BA 22, 37, 39	Group differences = NR *t* = NR, *p* = 0.311	Group differences = NR *t* = NR, *p* = 0.127	*F* = NR, *p* = 0.052	High active: 0.05 (0.26) Low active: 0.12 (0.49) *t* = NR, *p* > 0.01	High active: 0.32 (0.22) Low active: 0.09 (0.25) *t* = NR, *p* < 0.01
			BOLD response left BA 21	High active > low active *t* = NR, *p* = 0.001	e4+ > e4– *t* = NR, *p* = 0.005	*F* = NR, *p* = 0.675	High active: 0.14 (0.42) Low active: −0.12 (0.43) *t* = NR, *p* = NR	High active: 0.44 (0.24) Low active: 0.10 (0.37) *t* = NR, *p* = NR
			BOLD response right BA 18, 19	Group differences NR *t* = NR, *p* = 0.719	Group differences = NR *t* = NR, *p* = 0.063	*F* = NR, *p* = 0.112	High active: −0.30 (0.15) Low active: −0.19 (0.60) *t* = NR, *p* = NR	High active: 0.01 (0.37) Low active: −0.17 (0.17) *t* = NR, *p* = NR
			BOLD response left BA 18, 19	Group differences = NR *t* = NR, *p* = 0.232	Group differences = NR *t* = NR, *p* = 0.373	*F* = NR, *p* = 0.013	High active: −0.47 (0.31) Low active: 0.18 (1.23) *t* = NR, *p* < 0.01	High active: 0.13 (0.54) Low active: −0.10 (0.39) *t* = NR, *p* > 0.01
	Tsai et al. (2019)	${\text{VO}}_{2}^{\text{max}}$ (estimated from Rockport Fitness Walking Test)	EEG alpha band power 250–550 ms following stimulus onset	*r* = NR, *p* = NR	Group means = NR *t* = NR, *p* = NR	NA. Analyses stratified by *APOE*	*r* = NR, *p* > 0.05	*r* = NR, *p* > 0.05
	Tsai et al. (2021)	${\text{VO}}_{2}^{\text{max}}$ (estimated from Rockport Fitness Walking Test)	EEG P3 average amplitude between 300 and 650 ms (μV) following stimulus onset (memory non-switch condition)	*r* = NR, *p* > 0.05	Group means = NR *t* = NR, *p* > 0.05	NA. Analyses stratified by *APOE*	*r* = −0.06, *p* = 0.72	*r* = −0.26, *p* = 0.25
			EEG P3 average amplitude between 300 and 650 ms (μV) following stimulus onset (memory switch condition)	*r* = NR, *p* > 0.05	e4-: 7.98 (3.51) e4+: 5.03 (3.31) *t* = NR, *p* = 0.007	NA. Analyses stratified by *APOE*	*r* = 0.04, *p* = 0.78	*r* = −0.17, *p* = 0.46
			EEG P3 average amplitude between 300 and 650 ms (μV) following stimulus onset (number non-switch condition)	*r* = NR, *p* > 0.05	Group means = NR *t* = NR, *p* > 0.05	NA. Analyses stratified by *APOE*	*r* = 0.12, *p* = 0.44	*r* = −0.05, *p* = 0.83
			EEG P3 average amplitude between 300 and 650 ms (μV) following stimulus onset (number switch condition)	*r* = NR, *p* > 0.05	Group means = NR *t* = NR, *p* > 0.05	NA. Analyses stratified by *APOE*	*r* = 0.06, *p* = 0.68	*r* = −0.06, *p* = 0.80
Resting-state activation	de Frutos-Lucas et al. (2018)	High PA: ≥3 days vigorous PA totalling ≥1500 MET-min/week, or ≥7 days of any PA totalling ≥3000 MET-min/week (*n* = 16) Moderate PA: ≥3 days of ≥20 min PA per day, or ≥5 days walking for ≥30 min per day, or ≥5 days of any PA totalling ≥600 MET-min/week (*n* = 60) Low PA: not qualifying for moderate or high category (*n* = 24)	MEG Individual alpha peak frequency (Hz)	High: 9.97 (0.86) Moderate: 9.42 (0.82) Low: 9.22 (0.56) High v Mod, *t* = NR, *p* = 0.009 High v Low, *t* = NR, *p* =.002 Mod v Low, *t* = NR, *p* = 0.689	e4– > e4+ *F* = 5.993, *p* = 0.016	*F* = 0.531, *p* = 0.590 *F* = 4.882, *p* = 0.030^a^	High: 10.17 (0.92) Moderate: 9.51 (0.76) Low: 9.20 (0.56) High v Mod, *t* = NR, *p* = 0.005 High v Low, *t* = NR, *p* = 0.001 Low v Mod, *t* = NR, *p* = 0.714	High: 9.43 (0.29) Moderate: 8.89 (0.97) Low: 9.27 (0.63) High v Mod, *t* = NR, *p* > 0.9 High v Low, *t* = NR, *p* > 0.9 Low v Mod, *t* = NR, *p* > 0.9
	de Frutos-Lucas et al. (2020b)	Total PA from accelerometer	Average alpha band power during 5-min recording (MEG)	rho = 0.360, *p* < 0.001	Group means = NR, *t* = NR, *p* = NR	β = NR, *p* = 0.923	rho = 0.326, *p* = 0.004	rho = 0.442, *p* = 0.007
	Zlatar et al. (2014)	Total PA/hour calculated from the sum of the average number of minutes per hour of light PA ( ≤ 1,951 accelerometer counts), moderate PA (1,952–5,725 counts), and vigorous PA (≥5.726 counts)	ASL left hippocampus cerebral blood flow (mL/100 g tissue/min)	β = −0.1, *p* = 0.77	β = 0.1, *p* = 0.39	β = −0.4, *p* = 0.07	β = −0.061, *p* = 0.772	β = −0.705, *p* = 0.021
			ASL right hippocampus cerebral blood flow (mL/100 g tissue/minute)	β = 0.1, *p* = 0.65	β = 0.1, *p* = 0.60	β = −0.4, *p* = 0.07	β = 0.098, *p* = 0.649	β = −0.554, *p* = 0.068
Resting-state functional connectivity	de Frutos-Lucas et al. (2020a)	Total PA (accelerometer minutes from bouts of ≥10 min)	Strength of MEG oscillatory synchronicity between temporal lobe cluster and whole brain in the theta band	rho = −0.307, *p* = 0.0013	Group means = NR *t* = NR, *p* = NR	β = NR, *p* = 0.044	rho = −0.210, *p* = 0.0723	rho = −0.475, *p* = 0.0052
			Strength of MEG oscillatory synchronicity between temporal lobe cluster and whole brain in the delta band	rho = −0.361, *p* = 0.0001	Group means = NR *t* = NR, *p* = NR	β = NR, *p* = 0.13	rho = −0.301, *p* = 0.0091	rho = −0.458, *p* = 0.0074
			Strength of MEG oscillatory synchronicity between temporal lobe cluster and frontal/parietal lobe cluster in the delta band	rho = −0.425, *p* = 0.00001	Group means = NR *t* = NR, *p* = NR	β = NR, *p* = NR	rho = −0.353, *p* = 0.00203	rho = −0.612, *p* = 0.00025
			Strength of MEG oscillatory synchronicity between temporal lobe cluster and occipital lobe cluster in the delta band	rho = −0.440, *p* = 0.000001	Group means = NR *t* = NR, *p* = NR	β = NR, *p* = NR	rho = −0.423, *p* = 0.00017	rho = −0.610, *p* = 0.00016
	Kerestes et al. (2015)	Pedometer steps per week	fMRI functional connectivity between the ventral rostral posterior cingulate cortex and supplementary motor area	*r* = NR, *p* = NR	Group means = NR *t* = NR, *p* = NR	NA. Analyses stratified by *APOE*	*r* = NR, *p* > 0.05	*r* = 0.64, *p* = 0.001.

*APOE, Apolipoprotein E; ASL, Arterial spin labelling; BOLD, Blood oxygen level dependent; e4–, No APOE e4 alleles; e4+, Carrier of one or two APOE e4 alleles (includes e2e4 genotype unless stated otherwise); EEG, Electroencephalogram; fMRI, Functional magnetic resonance imaging; MEG, Magnetoencephalogram; MET, Metabolic equivalent of task; NA, Not applicable; NR, Not reported; PA, Physical activity. Additional data not included in the original publications are included in this review for Smith et al. (2011), Zlatar et al. (2014), de Frutos-Lucas et al. (2018, 2020a), and Tsai et al. (2021).*

a *ANCOVA model omitting main effects, including only the PA x APOE interaction term. Authors stated that low power to detect significant effect of the interaction term in the full model justified analysis of the interaction term alone to reduce chances of type 2 error; see de de Frutos-Lucas et al. (2018)*.

#### Task-Related and Resting-State Activity

**Figure 6:**
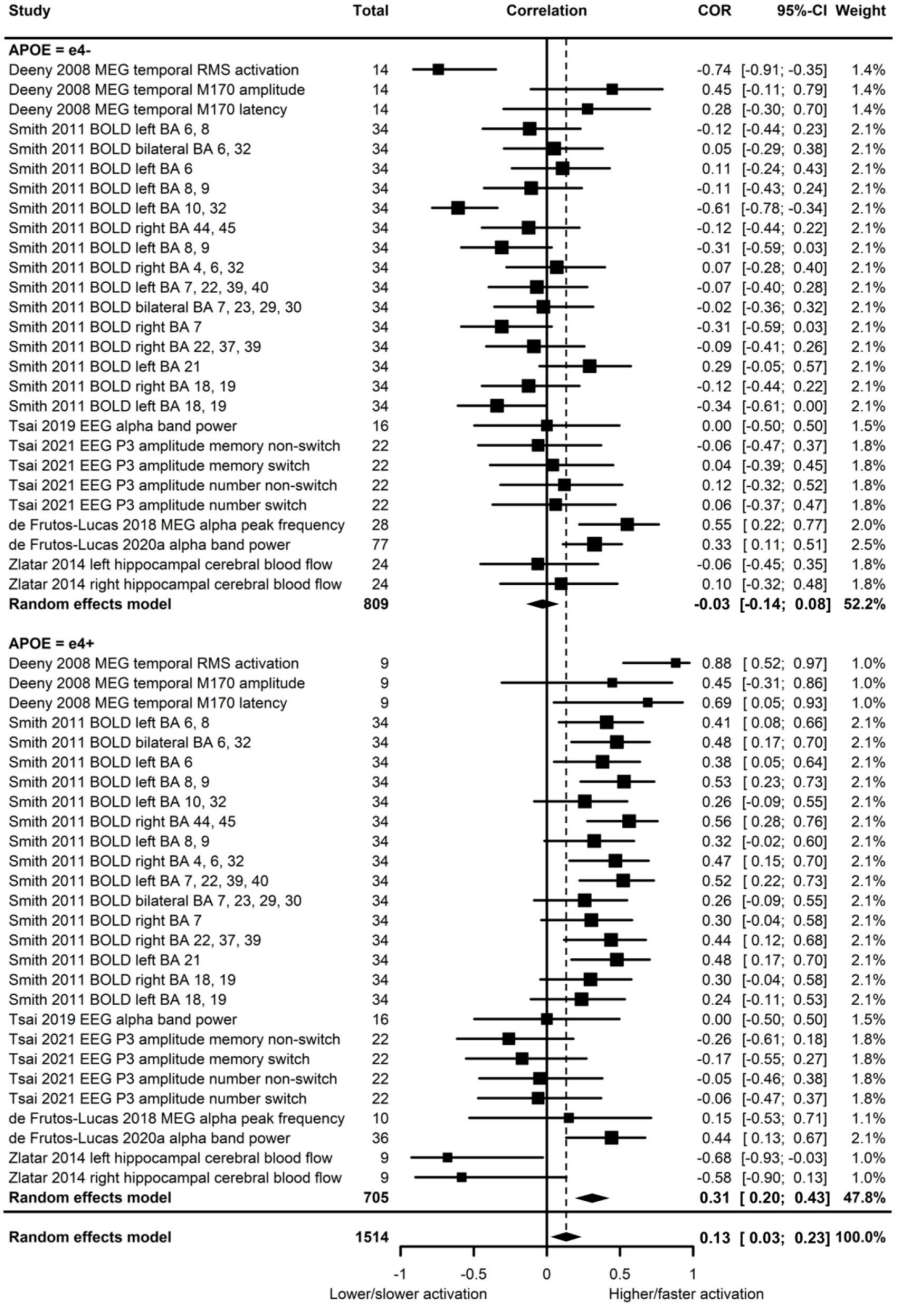
Forest plot indicating the association between physical activity and functional brain activation with carrier (e4+) and non-carrier (e4–) subgroups. Subgroup moderation test indicated a significant difference between *APOE* groups (*p* < 0.01).

Of the seven studies which assessed brain activation, four provided evidence of *APOE* moderation of the physical activity-brain activation association. A meta-analysis was carried out with 27 effect sizes each for e4 carriers and non-carriers, one of which was substituted with the main effect for e4 carriers and non-carriers combined, and one substituted with 0 due to the main effect not being reported. The multilevel model was not a significantly (*p* = 0.19) better fit than the standard model (see Supplementary Table 2 for model fit statistics). Overall, physical activity was significantly associated with brain activation (*r* = 0.13, *p* = 0.01). A moderation test indicated that the association between physical activity and brain activation was significantly different across *APOE* subgroups [*F*_(1, 52)_ = 18.03, *p* < 0.01]; subgroup analyses indicated that the association was significant for e4 carriers (*r* = 0.31, *p* < 0.01), but not non-carriers (*r* = −0.03, *p* = 0.58) (Figure 6). Heterogeneity was 52.8%. Visual inspection of the funnel plot did not suggest publication bias (Supplementary Figure 1).

For the studies which demonstrated significant physical activity by *APOE* interactions on brain activation, we considered whether there were differences in cognitive ability across *APOE* to assess if there was evidence of compensatory mechanisms in e4 carriers. In the study by Deeny et al. (2008), there was no difference between e4 carriers and non-carriers on the Cambridge Cognition Examination, nor on the working memory task used for MEG analysis. However, physical activity was associated with greater and faster neural activation in e4 carriers. In Smith et al. (2011), memory performance did not differ between e4 carriers and non-carriers, but physical activity was associated with increased BOLD activation more consistently in e4 carriers. In addition, spatial extent analysis indicated greater volume of activation in physically active e4 carriers only, and greater fMRI BOLD response in some regions indicated higher activation in e4 carriers. Zlatar et al. (2014) did not report cognitive differences across *APOE*, though the significant interaction between *APOE* and physical activity indicated the association between physical activity and resting-state cerebral blood flow was in e4 carriers only. However, the direction was reversed, with higher physical activity associated with lower cerebral blood flow.

#### Resting-State Functional Connectivity

Two studies assessed functional connectivity. de Frutos-Lucas et al. (2020a) assessed oscillatory synchronicity, which has been associated with dysfunction in AD. Physical activity was negatively associated with synchronicity, and while the association was consistently stronger in e4 carriers, only one of the four analyses demonstrated a significant physical activity by *APOE* interaction. Specifically, synchronicity between a temporal lobe cluster and the whole brain indicated that e4 carriers had an association between physical activity and reduced synchronicity (rho = −0.475, *p* < 0.01), but not non-carriers (rho = −0.210, *p* = 0.07). There were no differences in cognitive ability across *APOE*. Kerestes et al. (2015) investigated functional connectivity in the default mode network. Stratified analysis revealed a moderate association in e4 carriers (*r* = 0.64, *p* = 0.001), but no association in non-carriers. There were no differences in cognitive ability across *APOE*.

### Study Quality

**Figure 7:**
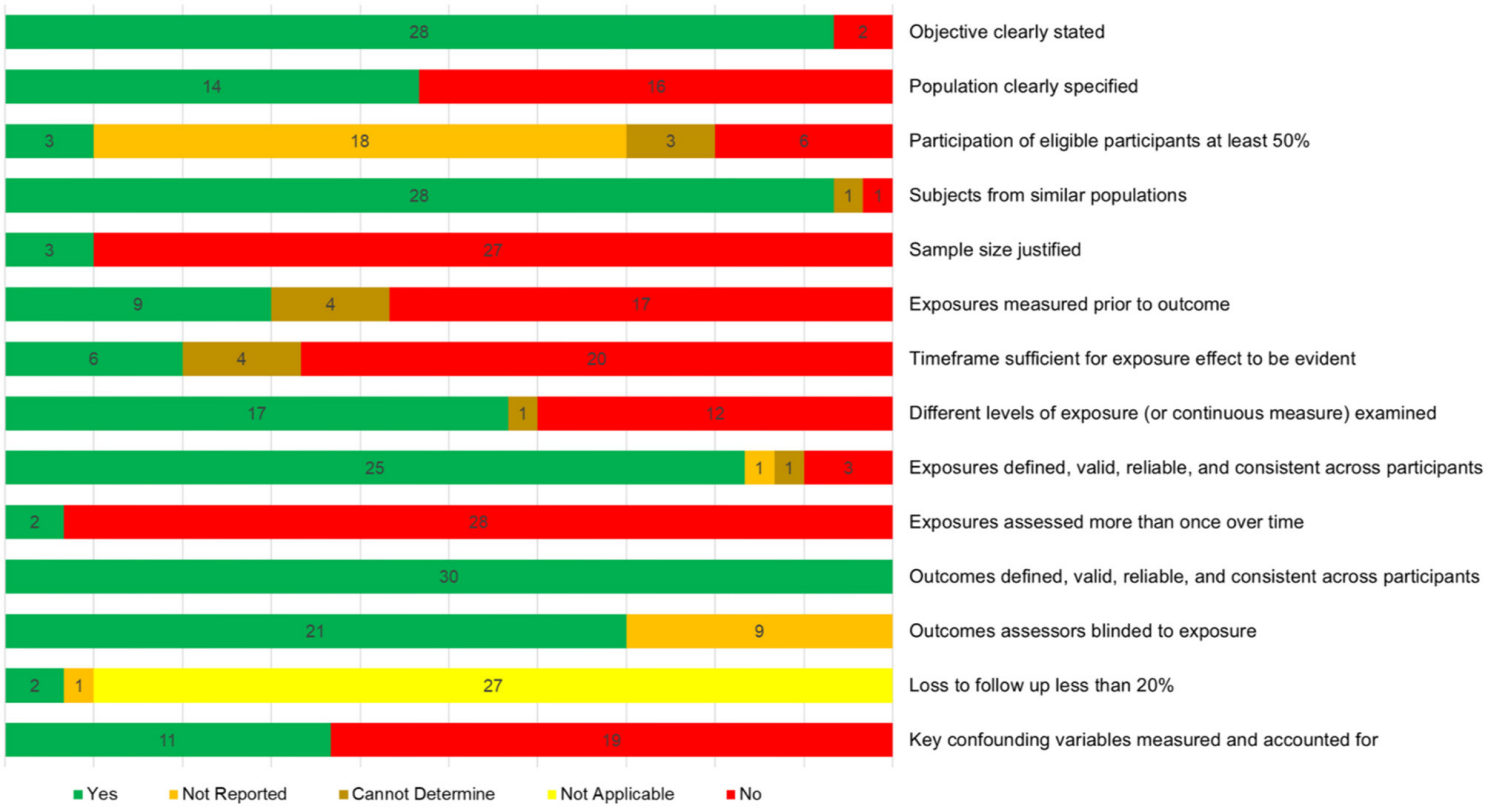
Quality assessment summary showing how many studies were given each of the five possible judgements for each of the assessment criteria.

Study quality judgements are shown in Supplementary Figure 2. None of the studies met/failed all four criteria (items 6, 7, 8, and 14) deemed essential for an overall judgement of good or bad, respectively, thus all studies were judged as fair overall. Figure 7 shows how many studies met each of the 14 criteria, demonstrating key areas for improvement. Only nine studies assessed physical activity levels prior to the outcome measurement, and only six of these allowed sufficient time for the effects of physical activity to be seen. Furthermore, only two studies measured physical activity over time. While three studies reported that participation rates were 50% or more, it was not possible to rate this for 21 studies. Finally, only 11 studies sufficiently controlled for potential confounds.

## Discussion

Meta-analyses indicated that physical activity was associated with better outcomes for HDL, GM and brain activation, but not for LDL and Aβ. Narrative syntheses revealed that one of three studies demonstrated an association between physical activity and WM volume (Gu et al., 2020); one study reported an association between physical activity and WM integrity (Smith et al., 2016); one of two studies demonstrated an association between physical activity and cerebrovascular health (Boots et al., 2015); and two of two studies reported an association between physical activity and functional connectivity (Kerestes et al., 2015; de Frutos-Lucas et al., 2020a).

In terms of *APOE* moderation, meta-analyses only indicated significant *APOE* differences in the association between physical activity and brain activation, with an association in e4 carriers but not non-carriers. Narrative syntheses provided some support for a difference in the association between physical activity and functional connectivity by *APOE* status. One study reported an association between physical activity and functional connectivity in both e4 carriers and non-carriers in three of the four analyses, and an association only in e4 carriers in the other analysis (de Frutos-Lucas et al., 2020a). The other study investigating functional connectivity found an association with physical activity only in e4 carriers (Kerestes et al., 2015).

### Lipid Profile

The meta-analyses indicated that physical activity was associated with HDL but not LDL, and no moderation by *APOE* for either. Publication bias was more likely for studies demonstrating a significant association with physical activity, but this pattern did not differ by *APOE* status. Assessment of TC was carried out narratively due to the complexity of interpreting TC levels. While one study (St-Amand et al., 1999) suggested *APOE* might moderate the association between physical activity and lipid profile, the other three studies (Boer et al., 1997; Bernstein et al., 2002; Pisciotta et al., 2003) did not. The one study (Boer et al., 1998) which looked at lipid risk profile as the outcome also suggested no moderating effect of *APOE*.

Overall, the results partially support the suggestion that physical activity benefits lipid profile. For HDL, the results indicate a beneficial association between physical activity and HDL, though this did not differ by *APOE* status. That is, those carrying the e4 allele are able to gain the same benefit from physical activity in terms of HDL levels as those without.

### Alzheimer's Disease Pathology

Based on the meta-analysis, physical activity was not associated with Aβ measured from PiB PET, CSF, blood plasma and erythrocytes, and the association did not differ by *APOE* status. Publication bias was as likely for e4 carriers and non-carriers, suggesting missing studies did not affect our ability to detect *APOE* differences. Although the results were consistent with higher levels of physical activity being associated with lower levels of Aβ, the overall association was not significant. This is supported by a recent review (Brown et al., 2019) which suggested that evidence for the association between physical activity and lower Aβ is more convincing in mice than in humans, and more work is needed to confirm whether physical activity is an effective means of reducing Aβ accumulation in humans.

### Brain Structure

The meta-analysis indicated that physical activity was significantly associated with GM volume. Interestingly, the subgroup analysis indicated that this association was only significant in e4 carriers, but the test of moderation was not significant (*p* = 0.06). There did not appear to be any publication bias, thus the overall association could be a reasonably accurate representation of the true effect. Indeed, there is evidence that physical activity and fitness is related to GM volume (Erickson et al., 2014), though further work is needed to confirm whether physical activity similarly benefits e4 carriers and non-carriers.

Only one study assessed WM integrity, demonstrating evidence of *APOE* differences in the association with physical activity (Smith et al., 2016). To accurately determine whether physical activity benefits WM integrity, it is important to note the limitations of WM integrity measurement. MD and FA measure the dispersion of water, which is used to infer the structural integrity of axons. However, in regions where axons cross, dispersion can appear high even when structural integrity is good (Pierpaoli and Basser, 1996; Madden et al., 2009). A greater understanding of the effect that crossing WM tracts have on measures of WM integrity would aid the interpretation of *APOE* differences in the association between physical activity and WM integrity, in addition to further studies simultaneously considering physical activity WM, and *APOE* status. With only one study, no firm conclusions can be made.

For cerebrovascular health, one of the two studies found evidence of an association between physical activity and WMH, but neither study provided evidence of a difference across *APOE*. Though there is evidence that physical activity does benefit cerebrovascular health (Wardlaw et al., 2015; Ritchie et al., 2017), there is no suggestion those benefits would differ by *APOE* status, albeit based on a limited number of studies.

### Brain Activation

For task-related and resting-state brain activation, the meta-analyses suggested that physical activity was associated with greater or faster brain activation in e4 carriers only. This effect appears to have been driven by two studies (Deeny et al., 2008; Smith et al., 2011), which contributed two-thirds of the effect sizes. Given that the model used for this analysis was not a multilevel model, the use of multiple effect sizes on the same participants might have spuriously indicated a significant effect in e4 carriers. However, when a multilevel random effects model was fitted to account for multiple effect sizes from each study, the result was unchanged (the multilevel model did not improve the model fit). The better fit of the standard model suggested that the multiple outcomes within a study were adding independent variance to the model.

*Post-hoc* investigation of the studies with *APOE* moderation revealed evidence that the association between physical activity and brain activation could be related to compensatory mechanisms in e4 carriers. Higher brain activation may be a mechanism through which the negative effect of e4 possession is masked. In a memory encoding task, a comparison of the blood oxygen level dependent (BOLD) response during the presentation of new pictures compared to a repeated picture facilitated an assessment of the “effort” needed to encode new memories. A greater BOLD response during memory encoding was seen in e4 carriers across occipital, parietal and frontal regions. However, with no difference in memory performance across *APOE* groups, it seems that e4 carriers “worked harder” to achieve comparable cognitive performance (Bondi et al., 2005). Brain activation when not engaged in a task also appears to show compensation for e4 possession. Resting-state cerebral blood flow was higher in e4 carriers, but there was no difference in brain activation during a memory task (Fleisher et al., 2009; Bangen et al., 2012). This upregulation of resting-state blood flow could enable sufficient cerebral blood flow during tasks in those with underlying neurological deficits, thus representing another potential compensatory mechanism.

Our meta-analysis indicated that physical activity was only associated with brain activation in e4 carriers, however, cognitive ability did not differ across *APOE* in the two studies which appeared to drive the effect (Deeny et al., 2008; Smith et al., 2011). Deeny et al. (2008) and Smith et al. (2011) both found physical activity to be associated with greater brain activation. Smith et al. (2011) also found evidence of greater brain activation in e4 carriers compared to non-carriers and suggested that physical activity could facilitate the neural upregulation necessary for e4 carriers to maintain cognitive ability during early neurodegeneration. If this suggestion is correct, it might be expected that active e4 carriers would show greater cognitive ability than inactive e4 carriers. This was the case for participants in the Deeny et al. (2008) study, but not in the Smith et al. (2011) study. Further studies are therefore required to determine whether and how physical activity might facilitate neural upregulation in e4 carriers, and the resultant effect on cognitive ability.

In contrast to the physical activity-related upregulation reported by Deeny et al. (2008) and Smith et al. (2011), Zlatar et al. (2014) demonstrated the opposite effect. In e4 carriers only, physical activity was associated with lower cerebral blood flow. As cognitive ability did not differ by *APOE*, Zlatar et al. (2014) interpreted these findings as demonstrating a compensatory mechanism in physically inactive e4 carriers, whereby resting-state cerebral blood flow was upregulated. This interpretation contradicts the suggestion that physical activity facilitates upregulation, instead implicating a lack of physical activity as a reason for upregulation becoming necessary. The association between physical activity and cognitive ability in e4 carriers was not reported, so it is not clear whether physical activity-related differences in cerebral blood flow influenced cognitive ability. Overall, our meta-analysis provides some support for the beneficial effect of physical activity in facilitating compensation in e4 carriers, but further studies are needed to confirm this given the limited number of studies available.

Functional connectivity was investigated in two studies, with both providing evidence for the association between physical activity and functional connectivity differing by *APOE*. One study found reduced oscillatory hypersynchrony to be associated with physical activity in both e4 carriers and non-carriers, though potentially stronger in carriers (de Frutos-Lucas et al., 2020a). The other found better functional connectivity to be associated with physical activity in e4 carriers only (Kerestes et al., 2015). No differences in cognitive ability across *APOE* in these studies again indicates a possibility of physical activity aiding e4 carriers to compensate for deficits. Compensation may differ from upregulation and involve structural differences which facilitate communication between different brain regions.

Though these two methods of compensation share similarities in facilitating brain activation which maintains cognitive ability during early neurodegeneration, they may differ in other ways. Upregulation of brain activation is achieved by increased blood flow during a task (Buckner et al., 1996), whereas enhanced functional connectivity may also require structural differences in the form of connexions between distinct brain regions (van den Heuvel and Hulshoff Pol, 2010). The evidence in this review does not provide support for a beneficial effect of physical activity on general brain health in e4 carriers but does provide some support for a beneficial effect of physical activity in promoting the required neural architecture (Kamijo et al., 2011) and task-related neural upregulation (Yu et al., 2021) to facilitate compensation which allow e4 carriers to maintain cognitive ability during the early stages of neurodegeneration. As this is based on a small number of studies, further research is needed to confirm and further elucidate these mechanisms.

### Study Quality

#### Heterogeneity

As expected, there was evidence of heterogeneity across the meta-analyses. For LDL, heterogeneity was high and all of the I^2^ variance was between clusters. As each cluster contained effect sizes which used the same measure of physical activity and the same measurement of LDL, the only possible source of heterogeneity within a cluster was gender, and four of the six studies reported effect sizes separately for male and female participants. The within cluster homogeneity suggested that gender was not a source of heterogeneity. One potential difference between clusters was the LDL measurement, but as the two LDL metrics used (mmol/L and mg/dL) can be directly converted, this was unlikely to have caused heterogeneity. As a sensitivity analysis with the only longitudinal study removed made little difference to the heterogeneity, physical activity appears to be the most likely source. The pattern of heterogeneity was similar for HDL, with high heterogeneity all at the between study level cluster again demonstrating that the physical activity measurement was the most likely cause.

Heterogeneity among studies assessing Aβ was high with most of this variance at the between cluster level. In contrast to the models for LDL and HDL, where the outcomes were unlikely to represent a potential source of heterogeneity, the Aβ model included different methods of measuring the outcome. As some studies used multiple outcome measures, these differences could be evident even within a cluster. However, the amount of within cluster variance was low with the majority between clusters, suggesting that the Aβ measurement method was not a substantial source of heterogeneity. While some of the between cluster heterogeneity could have been due to differences in the Aβ measurement, as there were different combinations of measurements in each cluster, it seems likely again that the biggest source of heterogeneity among the studies was the measurement of physical activity.

Though heterogeneity in the GM volume model was lower than for the lipid and Aβ analyses, there was still moderate heterogeneity, with most of this at the between study cluster level. *Post-hoc* analyses indicated the measurement of physical activity and study design as potential sources of heterogeneity. Removing the one longitudinal study made little difference, with moderate heterogeneity mostly at the between cluster level, again suggesting physical activity measurement as the main source of heterogeneity.

#### Study Quality Assessment

All studies were judged as fair following assessment with the National Heart, Lung and Blood Institute's Quality Assessment Tool for Observational Cohort and Cross-Sectional Studies. While a clear metric would have been desirable, this tool is only designed to be used as a guide to aid authors in making an overall quality judgement. One criterion which all studies met was using outcomes that were defined, valid and reliable. Given the objective nature of the measures used for outcomes, it is perhaps unsurprising that they did not appear to contribute heterogeneity to the analyses. Measures of the exposure, i.e., physical activity, were also generally good, with 25 of the 30 studies deemed to have used defined, valid and reliable measures. However, given that the measures of physical activity appeared to introduce substantial heterogeneity into the analyses, the use of a consistent tool for measuring physical activity would improve the literature.

It would also be desirable for future studies to assess physical activity multiple times prior to the outcome being measured and with sufficient time for any potential benefits to become evident. In addition, more detailed reporting of participation rates would allow stronger conclusions to be drawn on the representativeness of the results (albeit within the context of the specific samples). Finally, robust controlling for potential confounds would facilitate stronger conclusions that physical activity itself is beneficial after ruling out factors such as blood pressure and BMI.

*APOE* allele frequencies were generally poorly reported, with only three studies explicitly stating that frequencies did not deviate from the Hardy-Weinberg equilibrium (Corella et al., 2001; Pisciotta et al., 2003; Gustavsson et al., 2012). Given that some studies selected participants for analysis based on *APOE* status, it was not possible to determine whether the samples reflected a representative selection of participants in terms of e4 possession.

### Limitations

One limitation of this review is that all studies were observational, being either cross-sectional, retrospective cohort or prospective cohort studies. Randomised controlled trials would provide stronger evidence for a causal association between physical activity and brain health. A second limitation is that not all data were available for meta-analyses. While attempts were made to acquire the missing data and no eligible studies were omitted due to this, only the meta-analysis for Aβ did not contain any estimated data points. The conclusions drawn from the meta-analyses on LDL, HDL, GM volume and functional brain activation therefore include a degree of uncertainty.

It is also worth noting that most studies did not investigate allele dose. In smaller studies, this is not possible due to the low number of people carrying two e4 alleles. While combining heterozygotes and homozygotes is not problematic, it meant that it was not possible to consider whether physical activity differentially benefits homozygotes, who are at the highest genetic risk. In addition, many studies did not demonstrate a significant main effect of *APOE*, which might be expected. If any increased benefit from physical activity in e4 carriers is only seen in those who are experiencing the negative effects of e4 possession, then analysis on those who are yet to experience the negative effects may fail to identify an increased benefit of physical activity. The lower participation of individuals with poorer health, including Alzheimer's (Tyrrell et al., 2021) could potentially explain why no *APOE* effect was observed.

Finally, a common approach among studies in this review was to assess the association between physical activity and the outcome separately for e4 carriers and non-carriers. While this stratified approach helps to identify whether the association differs by *APOE*, it does not determine whether any observed difference is statistically significant.

### Future Directions

While there is some evidence for a greater benefit of physical activity in e4 carriers, this appears to be dependent upon the outcome being assessed. Our findings suggest a nuanced pattern where physical activity does not benefit e4 carriers differently for lipids, Alzheimer's disease pathology, GM volume, WM volume or cerebrovascular health, but might for functional brain outcomes. Future studies could focus on brain activation and brain structure which facilitates functional connectivity to consider whether physical activity allows e4 carriers to maintain cognitive ability during the early stages of neurodegeneration. If physical activity facilitates improved neural processing, it might be expected that e4 carriers would benefit more from physical activity on cerebrovascular health, which was not supported by the current analyses. With only two studies on this outcome, more are needed to consider this possibility. If physical activity benefits cerebrovascular health to a greater extent in e4 carriers, it would provide support for compensation by neural upregulation in e4 carriers. If e4 carriers do not benefit more, this could indicate that any apparent compensation is through structural changes which facilitate efficient communication between distinct brain regions.

Detecting subtle associations would be aided if future studies could reduce heterogeneity within the literature, for example by using objective measurements of physical activity such as accelerometer data. Considering how best to measure physical activity would facilitate an exploration of whether findings differ based on self-report compared to objective measures, ultimately determining whether future studies should focus exclusively on objective measures. Furthermore, measures of physical fitness and fitness-related health measures could elucidate specific biological outcomes related to being physically active that are involved in any mechanism through which e4 carriers benefit from physical activity.

Future studies could also compare analyses in those already showing evidence of age-related decline to those who are not to see if any greater benefit from physical activity in e4 carriers is only seen in those who need to compensate. Analysis of the interaction between physical activity and the outcome would allow a judgement on whether the association is significantly different in e4 carriers compared to non-carriers. Finally, analysis in large scale datasets where there are enough e4 homozygotes could uncover whether there is a difference in the benefit gained from physical activity in those at the highest genetic risk.

## Conclusion

The current review indicates that those carrying the *APOE* e4 allele gain at least the same benefit from physical activity as those without. There is tentative support that the benefit of physical activity might be greater for e4 carriers specifically in relation to brain activation. However, the evidence is limited and further research is required to confirm this.

## Data Availability Statement

The datasets presented in this article are not readily available because the results were drawn from published studies for inclusion in the systematic review, and where relevant, meta-analyses. All data relevant to the analyses are presented within the manuscript so no additional data posting is necessary. Requests to access the datasets should be directed to Alan J. Gow, a.j.gow@hw.ac.uk.

## Author Contributions

AP, MD, and AG contributed to conception and design of the study. AP developed the systematic review protocol, with input from MD and AG. AP conducted the systematic review with support from CM on study screening and reviewing. AP performed the statistical analysis and wrote the first draft of the manuscript. All authors contributed to manuscript revision, read, and approved the submitted version.

## Funding

The work was supported by a PhD Scholarship from the Centre for Applied Behavioural Sciences at Heriot-Watt University.

## Conflict of Interest

The authors declare that the research was conducted in the absence of any commercial or financial relationships that could be construed as a potential conflict of interest.

## Publisher's Note

All claims expressed in this article are solely those of the authors and do not necessarily represent those of their affiliated organizations, or those of the publisher, the editors and the reviewers. Any product that may be evaluated in this article, or claim that may be made by its manufacturer, is not guaranteed or endorsed by the publisher.
