# Supplementary material for: Apolipoprotein E Genotype Moderation of the Association Between Physical Activity and Brain Health. A Systematic Review and Meta-Analysis
Source: Front Aging Neurosci. 2022 Jan 28;13:815439. doi: 10.3389/fnagi.2021.815439 (PMC8833849; doi:10.3389/fnagi.2021.815439)
Supplement: Supplementary file 1 [file Table_1.docx]

Supplementary Table 1. Search terms

| Category | Physical activity | *APOE* | Outcomes | | | |
| --- | --- | --- | --- | --- | --- | --- |
|  |  |  | Lipids | AD pathology | Brain structure | Brain activation |
| Search terms | “physical activity”  “exercise”  “sedentary”  “acceleromet*”  “walk”  “walking”  “jog”  “jogging”  “run”  “running”  “cycle”  “cycling”  “swim”  “swimming”  “tennis”  “badminton”  “resistance train*”  “weight train*”  “gym”  “danc*”  “yoga”  “pilates”  “tai chi”  “active occupation”  “active job” | “apolipoprotein E”  “APOE”  “APOE4”  “apo E”  “apo E4”  “apo Ee4”  “apo E e4”  “apoEe4”  “e4”  “e3”  “e2”  “epsilon 4”  “epsilon 3”  “epsilon 2”  “rs429358”  “rs7412”  “dementia gene”  “Alzheimer’s gene”  “cognitive gene” | “cholesterol”  “lipoprotein”  “low density”  “high density”  “LDL”  “HDL”  “arterial”  “blood pressure” | “amyloid”  “tau”  “Pittsburgh compound-B”  “PiB”  “florbetapir” | “MRI”  “magnetic resonance imaging”  “grey matter volume”  “atrophy”  “cortical thickness”  “fractional anisotropy”  “FA”  “mean diffusivity”  “MD”  “white matter integrity”  “myelin*”  “tract”  “white matter hyperintens*”  “cerebrovascular”  “brain health” | “fMRI”  “functional connectivity”  “BOLD”  “blood oxygen level dependent”  “EEG”  “electroencephalography”  “MEG”  “magnetoencephalography“  “alpha peak”  “event related potential*” |

*Notes. APOE* = Apolipoprotein E; BOLD = Blood oxygenation level dependent; EEG = Electroencephalography; fMRI = Functional magnetic resonance imaging; FA = Fractional anisotropy; HDL = High density lipoprotein; LDL = Low density lipoprotein; MD = Mean diffusivity; MEG = Magnetoencephalography; MRI = Magnetic resonance imaging; PiB = Pittsburgh compound B. The overall search term was constructed by combining individual terms with Boolean operators. Three separate terms for physical activity, *APOE,* and outcomes were created within brackets with individual terms separated by ‘OR’. These three bracketed terms were then combined with ‘AND’. This resulted in a search term which returned papers which included at least one term from each section (physical activity, *APOE* and outcomes). An example including the first two terms from each column is: (“physical activity” OR exercise) AND (”apolipoprotein E” OR APOE) AND (cholesterol OR lipoprotein OR amyloid OR tau OR MRI OR “magnetic resonance imaging” OR fMRI OR “functional connectivity
