# Supplementary material for: Apolipoprotein E Genotype Moderation of the Association Between Physical Activity and Brain Health. A Systematic Review and Meta-Analysis
Source: Front Aging Neurosci. 2022 Jan 28;13:815439. doi: 10.3389/fnagi.2021.815439 (PMC8833849; doi:10.3389/fnagi.2021.815439)
Supplement: Supplementary file 2 [file Table_2.doc]

Supplementary Table 2. Model fit statistics for meta-analyses

| Meta-analysis | Total heterogeneity (I^2^) % | Within study cluster heterogeneity (I^2^) % | Between study cluster heterogeneity (I^2^) % | Akaike Information Criterion (AIC) | | Bayesian Information Criterion (BIC) | | Comparison of full versus reduced model (likelihood ratio chi-squared test p-value) |
| --- | --- | --- | --- | --- | --- | --- | --- | --- |
|  |  |  |  | Multilevel model | Model with study level removed | Multilevel model | Model with study level removed |  |
| LDL multilevel | 84.51 | 0 | 84.51 | -20.68 | -16.67 | -17.12 | -14.00 | .014 |
| HDL multilevel | 77.54 | 0 | 77.54 | -12.47 | -9.64 | -9.38 | -7.32 | .028 |
| Aβ multilevel | 86.50 | 6.48 | 80.02 | 3.76 | 9.04 | 8.47 | 12.58 | .007 |
| GM multilevel | 54.70 | 5.62 | 49.08 | -74.24 | -66.5 | -66.76 | -60.96 | .002 |
| Functional non-multilevel | 52.81 | 52.81 | - | 40.54 | 40.26 | 48.34 | 46.11 | .190 |
| Functional (task-related only) non-multilevel | 38.90 | 38.90 | - | 23.15 | 22.09 | 30.29 | 27.44 | .335 |

*Notes.* Aβ = Amyloid beta; GM = Grey matter; HDL = High density lipoprotein; LDL = Low density lipoprotein. Significantly lower AIC and BIC indicate superior model fit.
