# Supplementary material for: Apolipoprotein E Genotype Moderation of the Association Between Physical Activity and Brain Health. A Systematic Review and Meta-Analysis
Source: Front Aging Neurosci. 2022 Jan 28;13:815439. doi: 10.3389/fnagi.2021.815439 (PMC8833849; doi:10.3389/fnagi.2021.815439)
Supplement: Supplementary file 3 [file Data_Sheet_1.doc]

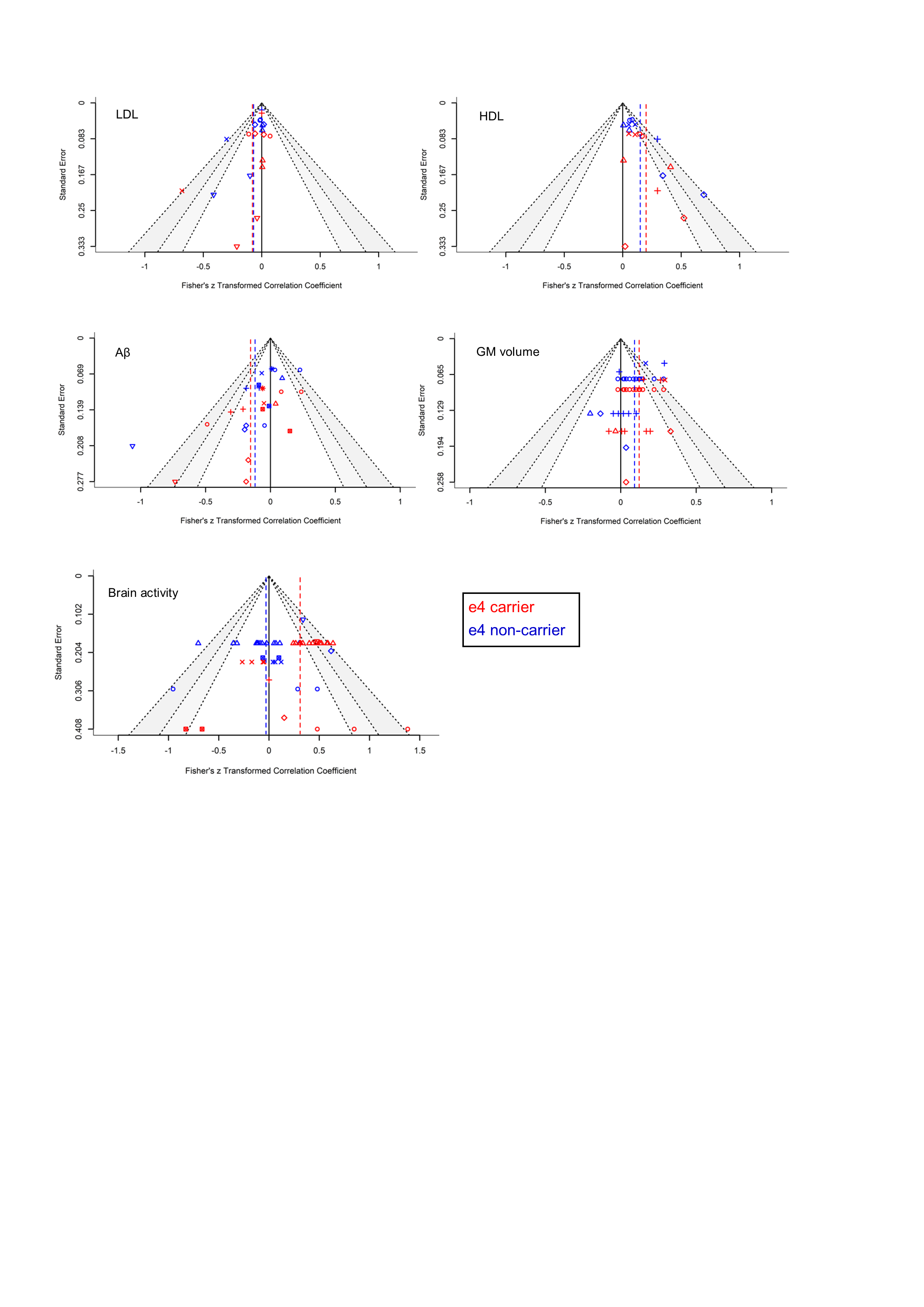


Supplementary Figure 1. Funnel plots for each meta-analysis.

*Note.* Effect sizes are plotted on the x axis against standard error, an indicator of the accuracy of the effect size, on the y axis.
