# Supplementary material for: Apolipoprotein E Genotype Moderation of the Association Between Physical Activity and Brain Health. A Systematic Review and Meta-Analysis
Source: Front Aging Neurosci. 2022 Jan 28;13:815439. doi: 10.3389/fnagi.2021.815439 (PMC8833849; doi:10.3389/fnagi.2021.815439)
Supplement: Supplementary file 4 [file Data_Sheet_2.doc]

|  | Objective clearly stated | Population clearly specified | Participation of eligible participants at least 50% | Subjects from similar population | Sample size justified | Exposures measured prior to outcome | Timeframe sufficient for exposure effect to be evident | Different levels or exposure (or continuous measure) examined | Exposures defined, valid, reliable, and consistent across participants | Exposure assessed more than once over time | Outcomes defined, valid, reliable, and consistent across participants | Outcome assessors blinded to exposure | Loss to follow up less than 20% | Key confounding variables measured and adjusted | Overall judgment of study quality |
| --- | --- | --- | --- | --- | --- | --- | --- | --- | --- | --- | --- | --- | --- | --- | --- |
| Bernstein et al., 2002 | Yes | Yes | Yes | Yes | No | No | No | Yes | Yes | No | Yes | Yes | NA | Yes | Fair |
| Boer et al., 1997 | Yes | Yes | No | Yes | Yes | CD | CD | CD | NR | No | Yes | Yes | NA | No | Fair |
| Boer et al., 1998 | Yes | Yes | No | Yes | No | CD | CD | No | Yes | No | Yes | Yes | NA | Yes | Fair |
| Boots et al., 2015 | Yes | Yes | CD | Yes | No | No | No | Yes | Yes | No | Yes | Yes | NA | No | Fair |
| Brown et al., 2013 | Yes | Yes | CD | Yes | No | Yes | No | Yes | Yes | No | Yes | Yes | NA | No | Fair |
| Corella et al., 2001 | Yes | Yes | Yes | Yes | No | No | No | No | Yes | No | Yes | Yes | NA | Yes | Fair |
| de Frutos-Lucas et al., 2018 | Yes | No | NR | Yes | Yes | No | No | Yes | Yes | No | Yes | NR | NA | No | Fair |
| de Frutos-Lucas et al., 2020a | Yes | No | NR | Yes | No | No | No | Yes | Yes | No | Yes | NR | NA | Yes | Fair |
| de Frutos-Lucas et al., 2020b | Yes | No | NR | Yes | No | No | No | Yes | Yes | No | Yes | NR | NA | Yes | Fair |
| de Souto Barreto et al., 2015 | Yes | No | NR | Yes | No | No | No | Yes | CD | No | Yes | Yes | NA | Yes | Fair |
| Deeny et al., 2008 | Yes | No | NR | Yes | No | No | No | No | Yes | No | Yes | NR | NA | No | Fair |
| Gu et al., 2020 | Yes | Yes | No | Yes | No | No | No | Yes | Yes | No | Yes | Yes | NA | No | Fair |
| Gustavsson et al., 2012 | Yes | Yes | CD | Yes | No | CD | CD | No | No | No | Yes | Yes | NA | Yes | Fair |
| Head et al., 2012 | Yes | Yes | NR | Yes | No | Yes | Yes | No | Yes | No | Yes | Yes | NA | Yes | Fair |
| Honea et al., 2009 | Yes | Yes | NR | Yes | No | Yes | No | Yes | Yes | No | Yes | Yes | NA | No | Fair |
| Jeon et al., 2020 | Yes | Yes | NR | Yes | No | Yes | Yes | Yes | Yes | No | Yes | Yes | NA | No | Fair |
| Kerestes et al., 2015 | Yes | Yes | NR | Yes | No | No | No | Yes | Yes | No | Yes | NR | NA | No | Fair |
| Liang et al., 2010 | No | No | NR | Yes | Yes | Yes | Yes | No | Yes | No | Yes | Yes | NA | No | Fair |
| Piccarducci et al., 2019 | Yes | No | NR | Yes | No | CD | CD | No | Yes | No | Yes | Yes | NA | No | Fair |
| Pisciotta et al., 2003 | Yes | No | NR | No | No | No | No | No | No | No | Yes | Yes | NA | Yes | Fair |
| Schmitz et al., 2001 | Yes | Yes | NR | Yes | No | No | No | Yes | Yes | Yes | Yes | Yes | Yes | Yes | Fair |
| Smith et al., 2011 | Yes | No | No | Yes | No | No | No | No | Yes | No | Yes | NR | NA | No | Fair |
| Smith et al., 2014 | Yes | No | No | Yes | No | Yes | Yes | No | Yes | No | Yes | Yes | Yes | No | Fair |
| Smith et al., 2016 | Yes | No | No | Yes | No | No | No | No | Yes | No | Yes | Yes | NA | No | Fair |
| St.-Amand et al., 1999 | Yes | No | NR | Yes | No | No | No | Yes | Yes | No | Yes | Yes | NA | No | Fair |
| Stojanovic et al., 2020 | Yes | No | NR | Yes | No | Yes | Yes | No | Yes | Yes | Yes | Yes | NA | Yes | Fair |
| Tsai et al., 2019 | Yes | No | NR | Yes | No | No | No | Yes | Yes | No | Yes | NR | NA | No | Fair |
| Tsai et al., 2021 | No | No | NR | Yes | No | No | No | Yes | Yes | No | Yes | NR | NA | No | Fair |
| Vemuri et al., 2016 | Yes | Yes | Yes | CD | No | Yes | Yes | Yes | No | No | Yes | Yes | NR | No | Fair |
| Zlatar et al., 2014 | Yes | No | NR | Yes | No | Yes | No | Yes | Yes | No | Yes | NR | NA | No | Fair |

Supplementary Figure 2. Quality assessment judgements for each study using the NHLBI quality assessment tool for observational cohort and cross-sectional studies.
